# Stimuli-Responsive Polymers for Advanced ^19^F Magnetic Resonance Imaging: From Chemical Design to Biomedical Applications

**DOI:** 10.1021/acs.biomac.4c00833

**Published:** 2024-08-16

**Authors:** Tuba Ayça Tunca Arın, Ondrej Sedlacek

**Affiliations:** Department of Physical and Macromolecular Chemistry, Faculty of Science, Charles University, 128 00 Prague 2, Czech Republic

## Abstract

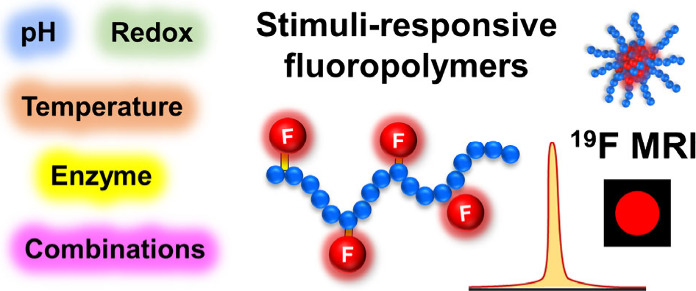

Fluorine magnetic resonance imaging (^19^F MRI) is a rapidly evolving research area with a high potential to advance the field of clinical diagnostics. In this review, we provide an overview of the recent progress in the field of fluorinated stimuli-responsive polymers applied as ^19^F MRI tracers. These polymers respond to internal or external stimuli (e.g., temperature, pH, oxidative stress, and specific molecules) by altering their physicochemical properties, such as self-assembly, drug release, and polymer degradation. Incorporating noninvasive ^19^F labels enables us to track the biodistribution of such polymers. Furthermore, by triggering polymer transformation, we can induce changes in ^19^F MRI signals, including attenuation, amplification, and chemical shift changes, to monitor alterations in the environment of the tracer. Ultimately, this review highlights the emerging potential of stimuli-responsive fluoropolymer ^19^F MRI tracers in the current context of polymer diagnostics research.

## 1 Introduction

Often termed “smart” polymers, stimuli-responsive polymers show high potential in biomedical sciences as an emerging class of materials.^1,2^ These materials stand out for their unique ability to change their physical properties, including water-solubility, in response to specific physicochemical stimuli, such as temperature, pH, light, and specific triggering molecules.^3^ Such polymers can then respond to either the internal biological environment or external physical triggers, thereby enhancing personalized healthcare tools, with potential applications in drug delivery,^4^ tissue engineering,^5^ diagnostics,^6^ and biosensing.^1^ Case in point, stimuli-responsive polymers are particularly suitable for developing intelligent materials for the diagnosis of diseases such as cancer.

In addition to their use as responsive carriers of diagnostic labels, stimuli-responsive polymers can be designed to strengthen or attenuate the diagnostic signal intensity for triggered “on/off” switchable diagnostic probes.^7^ This phenomenon is often used in fluorescently labeled systems in which a stimulus triggers a change in the probe’s fluorescence intensity due to fluorescence quenching.^8^ Similar principles can be leveraged in stimuli-responsive magnetic resonance imaging (MRI) diagnostics by changing chemical shifts or the magnetic relaxation of specific polymer segments.^7^ In comparison with other diagnostic methods, MRI has several advantages, such as high imaging contrast with no exposure to ionizing radiation.

In the last two decades, fluorine (^19^F) MRI has been widely studied as an appealing alternative to hydrogen (^1^H) MRI, commonly used in clinical practice.^9^ While ^1^H MRI plays a key role in clinical soft tissue imaging, omnipresent water and lipid molecules create an extensive hydrogen background, which adversely affects functional imaging with paramagnetic (mainly gadolinium or iron oxide-based) contrast agents.^10^ By contrast, the ^19^F nucleus, with its 100% natural abundance and absence in biological tissues, provides a clear and distinct signal for MRI, enabling precise tracking of polymer tracers within the body, also known as “hotspot imaging”.^11^ Furthermore, the gyromagnetic ratio of fluorine is similar to that of hydrogen, so fluorinated tracers can be visualized by clinical MRI instruments after minor hardware modifications.

Currently, the most widely studied ^19^F MRI tracers are perfluorocarbons (PFCs), such as perfluoro crown ethers (PFCEs).^12^ PFCEs show strong ^19^F MR signals and are both relatively biologically inert and nontoxic. However, PFCs are generally extremely hydrophobic, so they must be encapsulated into delivery vesicles to ensure their biological function in an aqueous environment and favorable pharmacokinetics. In turn, fluorinated polymer tracers (FPTs) show several benefits over small-molecule PFCs because their polymer structure, size, and architecture can be tailored to endow them with targeted physicochemical and biological properties.^13^ Therefore, combining stimuli-responsive polymers with ^19^F MRI offers a powerful tool for “smart” responsive diagnostics.

**Figure 1:**
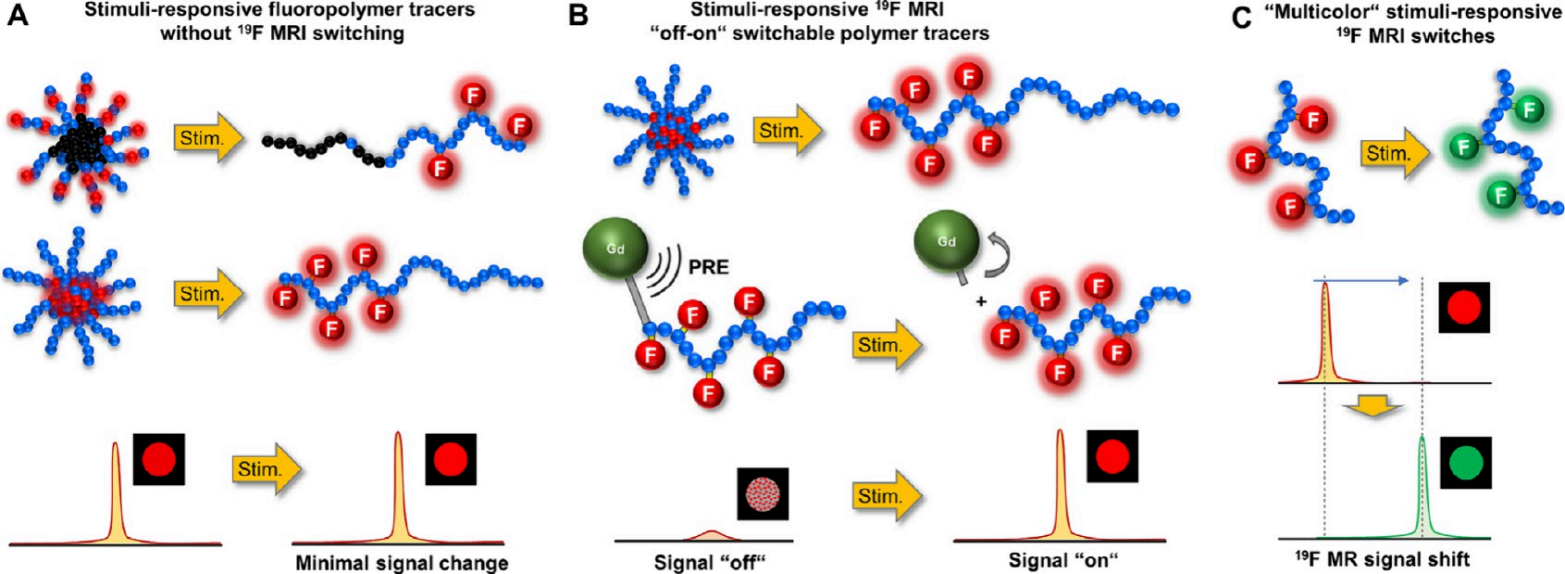
(A–C) Schematic illustration of three classes of stimuli-responsive fluoropolymer tracers for ^19^F MRI discussed in this review.

In this review, we provide a comprehensive overview of the state of the art stimuli-responsive fluorinated polymers applied as ^19^F MRI tracers. Several general reviews on stimuli-responsive ^19^F MRI tracers have been previously published,^14−16^ but they have focused on fluorine-loaded inorganic nanoparticles or on PFC encapsulated in nonfluorinated polymer nanoparticles, overlooking stimuli-responsive fluoropolymers, which are covered in depth in this review. Furthermore, we explore here the diverse range of stimuli that can be employed to trigger responses in polymers as well as various strategies for optimizing the interplay between fluorine content, water solubility, MRI properties, and stimuli responsiveness. More specifically, we discuss three types of stimuli-responsive fluorinated polymer tracers (Figure 1): (1) Stimuli-responsive fluoropolymer tracers, where the stimuli do not display substantial changes in MR signal shift or intensity in response to stimuli, the polymer is used as a responsive carrier for fluorine atoms, and the stimulus is used to trigger the change in carrier properties (e.g., self-assembly or disassembly). (2) Stimuli-responsive fluoropolymer tracers that show a significant change in ^19^F MRI signal intensity upon stimuli, so such “on/off” switchable tracers can be used to visualize pathological processes. (3) Stimuli-responsive fluoropolymer tracers with triggered changes in MR chemical shifts (often termed multicolor tracers) precisely report physicochemical stimuli. Lastly, we address challenges and future directions of the field of noninvasive medical diagnostics, highlighting the potential of smart fluoropolymer tracers to revolutionize this rapidly evolving diagnostic field.

## 2 Fluorinated Polymer Tracers for ^19^F MRI

The versatility of fluorinated polymer MRI tracers opens up numerous possibilities regarding the polymer chemical composition and chain architectures. In the past decade, considerable research efforts have been made to optimize the structure of ^19^F MRI tracers to combine excellent imaging sensitivity with desirable physicochemical and biological properties, such as water solubility, biocompatibility, and a favorable pharmacokinetic profile.^9^ However, the quality of the ^19^F MR signal is dependent on numerous parameters. In addition to hardware and measurement parameters, such as magnet strength, coil construction, echo (TE), and repetition time (TR), the sensitivity of the fluorinated tracer mainly depends on the fluorine content and its relaxation. Not only a high fluorine content but also optimal magnetic relaxation of fluorine atoms are necessary for high-intensity ^19^F MR images. The spin–lattice relaxation times (*T*_1_) should ideally be short to expedite *in vivo* image acquisition, but the spin–spin relaxation times (*T*_2_) should be long enough to achieve high-quality images by commonly used spin–echo-based MR sequences without requiring very short TE methods.

Broadly speaking, fluorinated polymer tracers can be divided into two classes based on the fluorinated chain solvation, namely (1) hydrophilic fluorinated polymers with well-solvated fluorinated chains, either as semifluorinated homopolymers or as statistical copolymers of fluorinated monomers with hydrophilic nonfluorinated comonomers, and (2) hydrophobic fluorinated polymers with high chain flexibility and low glass transition temperature (e.g., perfluoropolyethers), which are mainly used as core-forming blocks in self-assembled block copolymer nanoparticles. While the former often outperforms the latter in fluorine relaxation, their fluorine contents are slightly limited (up to approximately 25 wt %). Conversely, hydrophobic fluorinated polymers have high fluorine loadings but often inferior relaxation times due to the restricted mobility of self-assembled fluorinated segments.

**Figure 2:**
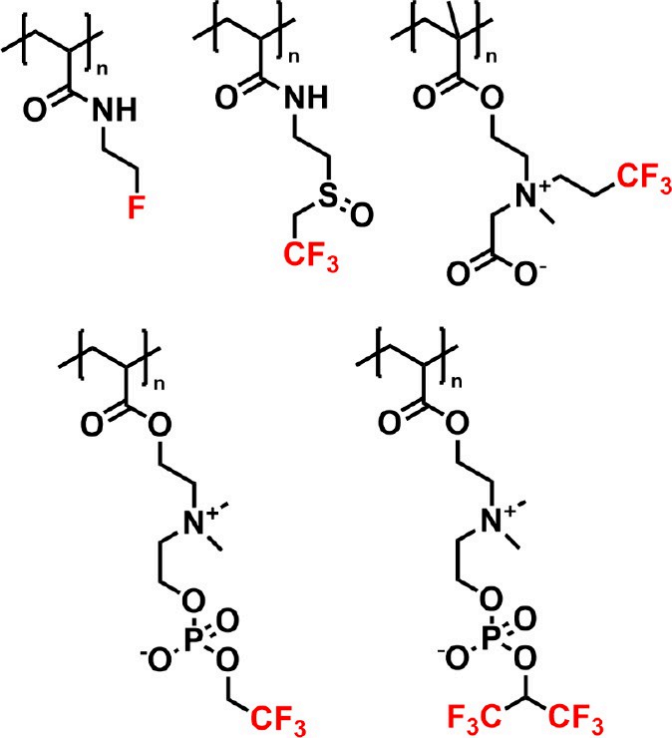
Representative examples of water-soluble fluorinated homopolymers studied as ^19^F MRI tracers.

Contrasting with extensive research on hydrophobic fluorinated homopolymers (e.g., polytetrafluoroethylene), reports on water-soluble fluorinated homopolymers remain scarce. In a monomer structure, the extremely hydrophobic fluorine must be outweighed by hydrophilic functional groups, such as amide, alcohol, sulfoxide, or charged groups (Figure 2). And to further boost monomer hydrophilicity, all unnecessary carbon atoms are removed (by replacing methacrylamides with more hydrophilic acrylamides, for example). Along these lines, Jirak and colleagues developed ^19^F MRI tracers based on water-soluble poly(*N*-(2-fluoroethyl)acrylamide) (PFEAM) with good MRI sensitivity and excellent antifouling properties.^17^ These ^19^F MRI tracers were therefore used as a hydrophilic coating for gold nanoparticles visualizable by MRI, but their ^19^F MRI performance was relatively limited due to their inherent fluorine signal splitting by two geminal hydrogens in the −CH_2_F group, leading to undesirable fluorine signal broadening.

Enhanced MRI performance can be expected from homopolymers containing trifluoromethyl groups, which provide a singlet MR signal. However, all three magnetically equivalent fluorine atoms must be outweighed by a higher number of hydrophilic functional groups. For example, Whittaker and colleagues synthesized a ^19^F MRI tracer consisting of water-soluble sulfoxide-containing poly(*N*-(2-((2,2,2-trifluoroethyl)sulfinyl)ethyl)acrylamide) (PFSAM) with a relatively high fluorine content (25 wt %) and an excellent *T*_2_ relaxation time of approximately 400 ms, which translated into an outstanding ^19^F MRI intensity.^18^ This polymer was linked to bovine serum albumin, and the resulting conjugate was successfully visualized by ^19^F MRI in mice.

Very high fluorine loading can be achieved in water-soluble fluorinated polyzwitterions, as shown by Huang and colleagues, who synthesized a carboxybetaine-based fluoropolymer to visualize HepG2 tumors in mice by ^19^F MRI.^19^ Similarly, Emrick and colleagues developed several fluorinated choline phosphate polyzwitterions, which showed good water solubility, cytocompatibility, and excellent antifouling properties.^20,21^ Acrylate-based fluoropolymers showed better ^19^F MR properties than their methacrylate-based analogs, enabling ^19^F MRI visualization of polymers *in vitro*. Water-soluble fluorinated homopolymers have also been prepared by ring-opening metathesis polymerization of norbornene imide bearing a trifluoromethyl group attached via a tetra(ethylene glycol) spacer, followed by dihydroxylation of the olefinic product to increase its water solubility.^22^ Moreover, some of these studies revealed a surprising finding: partial fluorination of hydrophilic polymers can improve their antifouling properties despite increasing their hydrophobicity.^17,20^ In the near future, we can expect extensive research to understand the molecular mechanism of this phenomenon thoroughly.

The area of water-soluble fluorinated homopolymers remains relatively narrow, but water-soluble statistical copolymers of fluorinated monomers with hydrophilic comonomers are a straightforward route to water-soluble fluoropolymers with a myriad of comonomer combinations. The interplay between fluorine content, ^19^F MRI properties, and polymer water solubility can be precisely adjusted by fine-tuning the comonomer ratios and the structure of both comonomers. Most water-soluble fluorinated copolymers reported thus far are acrylic-based polymers; nevertheless, other polymer types, including fluorinated polypeptides and poly(2-oxazoline)s, have also been reported.^23^ Generally, increasing the hydrophilicity of a hydrophilic comonomer increases the fluorine content at which the copolymers remain well soluble.^24^ To find a rational explanation, Leibfarth and colleagues applied a machine-learning approach to optimize the structure of acrylate-based fluorinated copolymers through automated copolymer synthesis.^25^ The study demonstrated that the ^19^F MRI signal intensity is not linearly related to the fluorine content at higher fluoromonomer contents, given the strong dipolar coupling of the ^19^F spins from neighboring repeating units.^24^ So, while a high fluorine content is desired, the possible F–F quenching effect should be considered when optimizing a copolymer structure.

Hydrophobic fluorinated polymers can be applied as water-soluble ^19^F MRI tracers in the form of amphiphilic copolymer nanoparticles self-assembled in an aqueous environment. These systems can provide higher fluorine contents, but the self-assembly of hydrophobic fluorinated blocks in the nanoparticle core may lead to substantial ^19^F MRI signal attenuation due to restricted chain mobility and low *T*_2_. For instance, diblock copolymer nanoparticles of (polyethylene glycol)-*b*-(poly(*N*-(2,2,2-trifluoroethyl)acrylamide)) (PEG-*b*-PTFEAM) nanoparticles directly synthesized by polymerization-induced self-assembly in water showed no ^19^F MR signal in an aqueous environment because their glassy PTFEAM core was rigid.^26^ This problem was eventually solved by incorporating a small amount of hydrophilic *N*-(2-hydroxyethyl)acrylamide into the hydrophobic block, leading to good ^19^F MRI images *in vivo*.^27^

In contrast to semifluorinated polyacrylamides, polymethacrylates (e.g., poly(*N*-(2,2,2-trifluoroethyl)methacrylate), PTFEMA) and, in particular, polyacrylates (e.g., poly(*N*-(2,2,2-trifluoroethyl)acrylate), PTFEA) show lower glass transition temperatures (*T*_g_) and can be visualized by ^19^F MRI. However, the incorporation of a plasticizing comonomer, such as *n*-butyl acrylate, significantly improves the MRI signal.^28,29^ Low-*T*_g_ perfluoropolyethers (PFPEs) also excel, even in a self-assembled state, due to their outstanding fluoropolymer chain mobility.^30^ Thanks to their high sensitivity, PFPE-based nanoparticles proved successful in the visualization of subcutaneous MDA-MB-468 tumors in murine models.^31^

**Figure 3:**
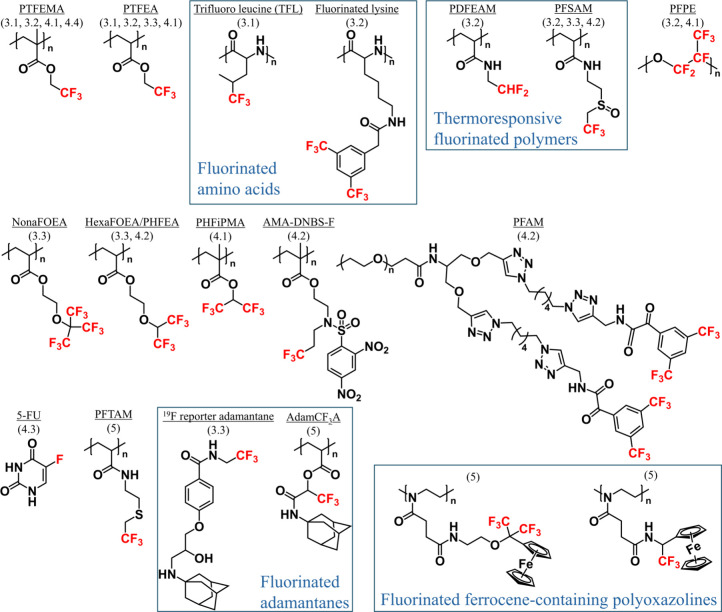
Structures of the fluorinated compounds discussed in this review. Related sections are given in the parentheses.

Strong fluoropolymer signal attenuation after self-assembly is undesirable for nonresponsive ^19^F MRI tracers. Nevertheless, such signal attenuation can be turned into profit in stimuli-responsive tracers with switchable ^19^F MR signals upon internal or external stimuli. These advanced systems are discussed in the following sections (Figure 3).

## 3 Stimuli-Responsive Fluoropolymer Tracers without ^19^F MRI Intensity Attenuation

Stimuli-responsive polymers show significant potential in biomedical sciences. Most prominently, a stimuli-responsive switch in polymer hydrophilicity can be utilized in the triggered self-assembly of the disassembly of amphiphilic copolymer nanoparticles or the change in hydrogel swelling. In drug-loaded nanoparticles, stimuli can trigger drug release. Various stimuli-responsive systems, such as polymers, have a lower critical solution temperature (LCST), which induces phase transition upon heating. In pH-responsive systems, upon protonation or deprotonation, responsive repeating units (amine and carboxylic acid) can become charged or oxidized by reactive oxygen species found in cancerous or inflamed tissue.

Following the fate of self-assembled polymers *in vivo* requires labeling these polymers with a tracing moiety such as a radioisotope, a fluorophore, or an MRI label. For ^19^F MRI, polymers must be labeled with magnetically relaxing fluorine atoms. However, the ^19^F MR intensity should not change with the stimulus in order to visualize the fluoropolymer after physicochemical transformation (e.g., self-assembly). In this chapter, we review such stimuli-responsive fluorinated tracers with none to a minor (<50%) ^19^F MR signal attenuation after phase transition (Figure 1A, Table 1). These fluoropolymers can then be used as ^19^F MRI tracers, even after triggering the physical change. In some cases, fluorinated moieties are merely added for imaging, and stimuli-responsive polymers are involved only in cargo delivery.

**Table 1:** Overview of Stimuli-Responsive ^19^F MR Tracers with None/Minor ^19^F Signal Response

stimulus	responsive unit	activation mechanism	^19^F MR response	ref
T	PFSAM	Assembly by LCST	Negligible	(18)
T	PDFEAM	Assembly by LCST	Attenuation	(38, 39)
T	PDFEAM	Assembly by LCST	Negligible	(40)
T	PFPE–POx	Assembly by LCST	Attenuation	(46)
T	POEGMA	Assembly by LCST	Attenuation	(48)
T	M-PEG	Assembly by LCST	Negligible	(49)
T	Fluorinated protein	Assembly by increased T	Negligible	(34)
T + ROS	PDFEAM + ferrocene	Assembly + disassembly	Negligible	(42)
T + pH	PDFEAM + imidazole	Assembly by LCST	Attenuation	(36)
pH	PAMAM dendrimer	Disassembly by protonation	Negligible	(52)
pH	THP	Hydrolysis of THP	Enhancement	(53)
pH	ZIF-8	Degradation of ZIF-8	Negligible	(57)
pH	Imine bonds	Release of DOX	N.A.^a^	(54)
pH/ion	PDMAEMA	Protonation of PDMAEMA	Negligible	(32)
Redox/pH	Disulfide/hydrazone	Hydrophobic drug release	Enhancement	(55)
pH	Sulfonamide	None (as a reporter)	No change	(66)
Redox	Disulfide	None (for biodegradability)	N.A.^a^	(33)
Oxygen	Fluorines	PRE effect	N.A.^a^	(61)
NaCl	POEGMA	Ionic hydration layer	Attenuation	(64, 65)

a Not reported.

### 3.1 Stimuli-Responsive Self-Assembled Nanoparticles with a Fluorinated Shell

^19^F MRI-traceable self-assembled nanoparticles can be easily prepared by introducing fluorine atoms into hydrophilic blocks of amphiphilic copolymers. The well-hydrated nanoparticle shell provides good magnetic relaxation of ^19^F nuclei, leading to excellent MRI signals. Concurrently, the maximal fluorine content is relatively limited to retain good water solubility of the hydrophilic block.

In 2020, Nurmi and colleagues prepared pH-responsive statistical (P(TFEMA-co-DMAEMA)) and block copolymers (PTFEMA-b-P(TFEMA-co-DMAEMA)) and (PTFEMA-b-P(TFEMA-co-METAI)) of trifluoroethyl methacrylate (TFEMA), pH-responsive 2-(dimethylamino)ethyl methacrylate (DMAEMA), and cationic [2-(methacryloyloxy)ethyl] trimethylammonium iodide (METAI) to assess the effect of solution conditions on conformations and ^19^F signal intensity.^32^ Because DMAEMA is a tertiary amine-containing monomer, its protonation depends on the pH. Thus, decreasing the pH increases the concentration of positively charged DMAEMA repeating units. By contrast, METAI is the quaternized form of DMAEMA and, as such, is positively charged at any pH. The statistical copolymers of the cationic monomers and fluorinated TFEMA prepared in this study were readily soluble in water, whereas the block copolymers self-assembled into nanoparticles with a glassy PTFEMA core and P(TFEMA-co-DMAEMA) or P(TFEMA-co-METAI) coronas. In turn, the statistical copolymers of TFEMA and DMAEMA were studied under various pH and ionic strength conditions, where the former had a significantly stronger effect on *T*_2_ relaxation in comparison to the latter.

Decreasing the pH increased the repulsion between the copolymers due to the increased number of positively charged units. This effect enhanced the mobility of fluorinated units and increased the *T*_2_ relaxation times. At low pH, copolymers of P(TFEMA-co-METAI) and P(TFEMA-co-DMAEMA) showed similar *T*_2_, and the pH did not affect the *T*_2_ times of P(TFEMA-co-METAI) because all of the repeating units were already cations. For the block copolymers, two *T*_2_ relaxation times were recorded, reflecting the two fluorinated populations, in the core and the corona. Due to the very short *T*_2_ relaxation times of the core, the effect of the highly fluorinated core was not detected on MRI images, so the results of the coronas matched those of statistical copolymers. All of these comparisons highlight the importance of fluorinated unit mobility for improving imaging performance.

**Figure 4:**
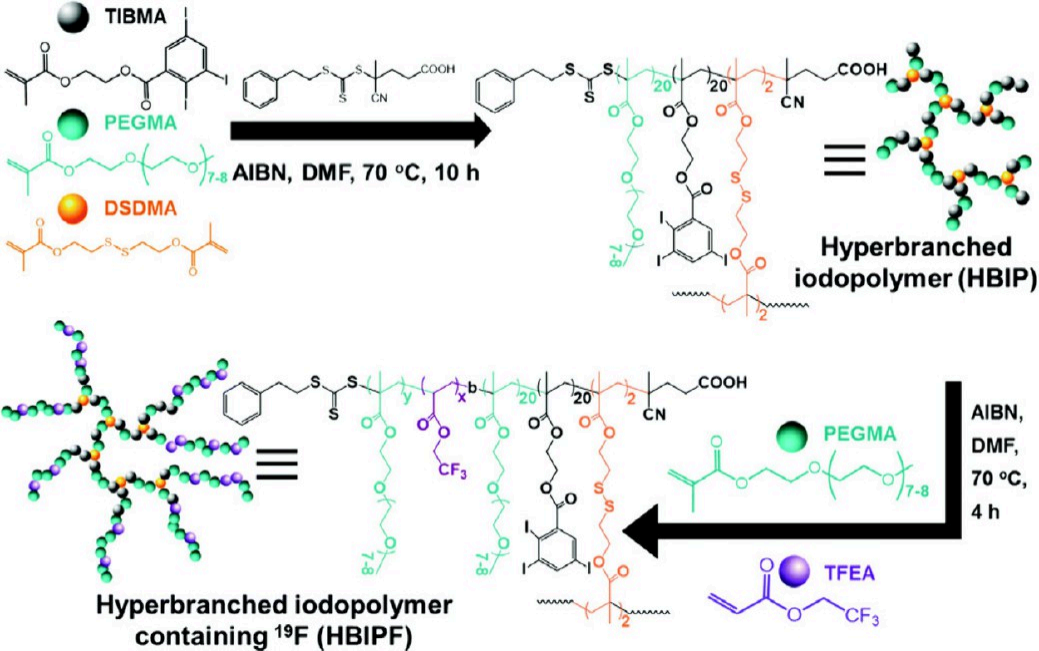
Redox-responsive hyperbranched fluoropolymers with disulfide-cross-linked core for CT/^19^F MRI bimodal molecular imaging. Reproduced with permission from ref (33). Copyright 2016, Royal Society of Chemistry.

Multimodal imaging probes can further enhance the potential of stimuli-responsive polymers for biomedical applications, as shown by Wang and colleagues, who developed a redox-responsive bimodal imaging system for both computed tomography (CT) and ^19^F MRI (Figure 4).^33^

For this purpose, they synthesized fluorinated hyperbranched iodopolymers (HBIPFs) by RAFT polymerization using a block copolymerization approach. First, they polymerized 2-(2′,3′,5′-triiodobenzoyl)ethyl methacrylate (TIBMA) – iodine-containing monomer (CT contrast agent), poly(ethylene glycol) methyl ether methacrylate (PEGMA) – hydrophilic monomer and bis(2-methacryloyl)oxyethyl disulfide (DSDMA) – redox responsive cross-linker into HBIP. Then, they synthesized the second block by copolymerization of 2,2,2-trifluoroethyl acrylate (TFEA) – fluorinated monomer (^19^F MRI agent) and PEGMA – hydrophilic monomer to enhance the fluorine mobility, yielding HBIPFs. Through this approach, they prepared three different polymers with varying fluorine content.

^19^F NMR characterization showed bimodal peaks, explained by differences in the chemical environments of the fluorines due to differences in the copolymerization reactivity of TFEA and PEGMA. Increasing fluorine content decreased both *T*_1_ and *T*_2_ relaxation times due to the reduced mobility of the fluorines. All polymers were tested in ^19^F MRI, providing clear images, especially at relatively high sample concentrations. The samples were also analyzed for their *in vivo* CT potential, and the results showed promise for bimodal imaging probes. Notwithstanding these advances, further research opportunities are still available as redox responsiveness was studied to assess the degradation of HBIPFs but not its impact on the ^19^F MRI signal intensity.

For ^19^F MRI tracing purposes, protein assembly can be induced by the temperature. By way of illustration, Hill and colleagues designed fluorinated thermoresponsive proteins (F-TRAPs), which assembled into nanosized micellar structures in response to an increase in temperature or concentration.^34^ These nanoassemblies were composed of a pentamer corona containing fluorinated trifluoroleucine (TFL) units and a hydrophobic, thermoresponsive polypeptide core. Temperature-driven micelle formation decreased the *T*_2_ relaxation time due to the formation of motionally restricted fluorines. These authors were able to overcome signal loss resulting from the decrease in *T*_2_ times, because they used zero echo time (ZTE) imaging. ^19^F ZTE MRI measurements resulted in less than 6% change in SNR upon a temperature increase. They used F-TRAP also for the delivery and release of a chemotherapeutic drug (doxorubicin, DOX), showing that increasing the temperature enhanced DOX release. More recently, the same research group published an article on protein-engineered nanofibers, which can act as temperature probes, outperforming their previous probes in ^19^F sensitivity and thermostability.^35^

### 3.2 Stimuli-Responsive Self-Assembled Nanoparticles with Hydrated Fluorinated Core

Self-assembly of fluorine-rich copolymers yields nanostructures with substantially attenuated ^19^F MRI intensity due to restricted chain mobility. In recent years, however, several thermoresponsive fluorinated polymers with LCST have shown only little ^19^F MRI signal attenuation after phase transition when heating over their cloud point temperature (*T*_CP_). Both examples, namely, poly(*N*-(2,2-difluoroethyl)acrylamide) (PDFEAM)^36^ and poly(*N*-(2-((2,2,2-trifluoroethyl)sulfinyl)ethyl)acrylamide) (PFSAM),^18^ share a balanced content of hydrophobic fluoroalkyl and hydrophilic groups (e.g., amide and sulfoxide group, respectively) within a single repeating unit.^18^ Such polymer chains presumably remain partly hydrated, even above their cloud point temperature.

Sulfoxide-containing thermoresponsive PFSAM fluoropolymers have been synthesized in Whittaker’s research group by controlled radical polymerization of the (*N*-(2-((2,2,2-trifluoroethyl)sulfinyl)ethyl)acrylamide) monomer.^18^ The polymer is water-soluble at room temperature, with LCST ranging from 30 to 40 °C, depending on the chain length and buffer. Even after heating above their *T*_CP_, the *T*_2_ relaxation times and ^19^F MRI signal-to-noise ratio remain excellent. Molecular dynamics simulations revealed significant temperature-induced dehydration of the backbone structure, leading to intermolecular aggregation, but the dehydration of the side-chain CF_3_ group was minimal, thanks to the adjacent sulfoxide group. This minimal CF_3_ dehydration was then reflected in the high segmental chain mobility and outstanding ^19^F MRI properties.

PDFEAM was first synthesized in 2012 by Bak and colleagues, who reported an LCST of approximately 25 °C.^37^ More recently, in 2018, Kolouchova and colleagues used PDFEAM as a thermoresponsive block in amphiphilic block copolymers with poly(*N*-(2-hydroxypropyl)methacrylamide) (PHPMA) or poly(2-methyl-2-oxazoline) (PMeOx) hydrophilic blocks.^38,39^ At room temperature, the copolymers were dissolved in water as individual chains. However, after heating over *T*_CP_ (approximately 30 °C, depending on copolymer composition), the fluorinated block became hydrophobic, self-assembling into nanoparticles with a hydrodynamic radius ranging from 30 to 80 nm. As shown by comprehensive light scattering, these large nanoparticles had a very low core density; therefore, they were classified as highly swollen physical nanogels.

These nanoparticles were then visualized by ^19^F MRI. After self-assembly above *T*_CP_, the ^19^F MR signal was only slightly attenuated but more so for PDFEAM than for PFSAM. Nevertheless, they were still traceable due to the high level of fluorine hydration. PDFEAM has another slight drawback in relation to PFSAM; its geminal hydrogen splitting in the CHF_2_ unit lowers the ^19^F MR signal amplitude. In turn, PDFEAM is more easily synthesized by single-step amidation from commercial starting materials.

The potential of thermoresponsive PDFEAM has been further exploited through the preparation of BAB triblock copolymers of PDFEAM-b-PEG-b-PDFEAM.^40^ At high temperatures, such copolymers self-assemble into either flower-like micelles or a physically cross-linked hydrogel, depending on copolymer structure and concentration. Again, the self-assembled fluoropolymer was successfully visualized by ^19^F MRI *in vitro*. PDFEAM has also been incorporated into gelatin hydrogels to induce hydrogel LCST when added at sufficient amounts (above 5% of DFEAM in the final hydrogel) and to enable hydrogel imaging by ^19^F MRI.^41^

**Figure 5:**
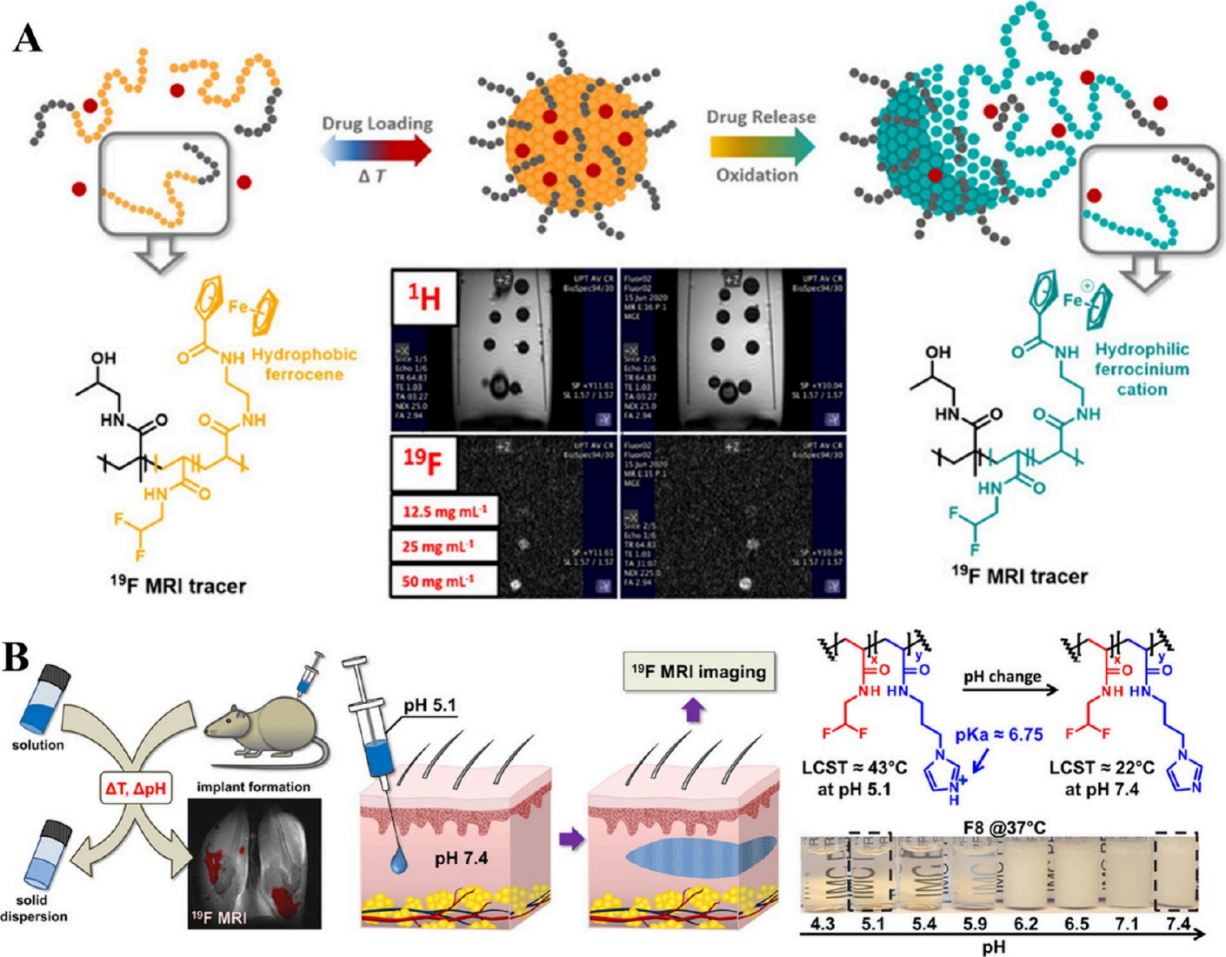
(A) Schematic illustration of dual temperature- and redox-responsive nanoparticles based on thermoresponsive PDFEAM. Reproduced with permission from ref (42). Copyright 2021, American Chemical Society. (B) Schematic illustration of the PDFEAM injectable implant formation, chemical structures, and stimuli response of fluorinated polymers. Reproduced with permission from ref (36). Copyright 2018, American Chemical Society.

While thermoresponsiveness generally derives from the in situ self-assembly of polymer nanoparticles, an additional responsive group is often added to ensure triggered nanoparticle disassembly and potential drug release in a targeted environment. Such additional stimuli can be, e.g., the acidic pH of a tumor, the inflammation site, and endolysosomes or reactive oxygen species (ROS). For instance, Kolouchova et al. prepared thermo- and ROS-responsive self-assembled polymer nanoparticle ^19^F MRI tracers by incorporating a ROS-responsive ferrocene moiety into the thermoresponsive block of the PHPMA-b-PDFEAM copolymer (Figure 5A).^42^ The copolymer self-assembled into nanoparticles at high temperatures, and the hydrophobic Fe^2+^ ferrocene group was oxidized to cationic Fe^3+^ ferrocenium by ROS, resulting in the nanoparticle core hydrophilization and disassembly. Yet, ^19^F MRI intensity was not affected by temperature-triggered self-assembly or by ROS-induced oxidation of diamagnetic Fe^2+^ ferrocene to paramagnetic Fe^3+^ ferrocenium. Finally, the nanoparticles were tested as DOX delivery systems.

Beyond its applications in block copolymer nanoparticles, thermoresponsive PDFEAM has been used to synthesize injectable implants traceable by ^19^F MRI (Figure 5B).^36^ The general strategy involved subcutaneous injection of an LCST polymer aqueous solution at room temperature (below the LCST). Upon heating to body temperature above LCST, the polymer becomes hydrophobic, thereby creating a solid implant, which avoids the need for surgical intervention. This method has a major disadvantage, though—rapid obstruction of the injection needle during the *in vivo* administration by phase-separated polymers.^43^

To overcome this drawback, Sedlacek et al. combined the thermoresponsiveness of PDFEAM with the pH-responsiveness of imidazole repeating units by statistical copolymerization of DFEAM with *N*-(3-imidazol-1-ylpropyl)acrylamide (ImPAM).^36^ The double-responsive fluorinated copolymer is injected into the body at a slightly acidic pH (approximately 5). At this pH, the imidazole group is positively charged, which increases the LCST above body temperature (43 °C) and eliminates the risk of needle obstruction. After injection, the copolymer is rapidly buffered to a physiological pH (7.4). As a result, imidazole is deprotonated, and LCST subsequently drops below the body temperature, which leads to phase separation of the copolymer into a solid implant.

Even after phase separation, the implant retains good ^19^F MRI traceability. The implant was visualized by ^19^F MRI in rats for several months before being cleared from the body. In a follow-up study, implant clearance was fine-tuned by incorporating a short segment of the hydrophilic *N*-(2-hydroxyethyl)acrylamide (HEAM) comonomer.^44^*In vivo* clearance of such implants was monitored by ^19^F MRI, showing faster clearance of copolymers with a higher HEAM content.^45^

**Figure 6:**
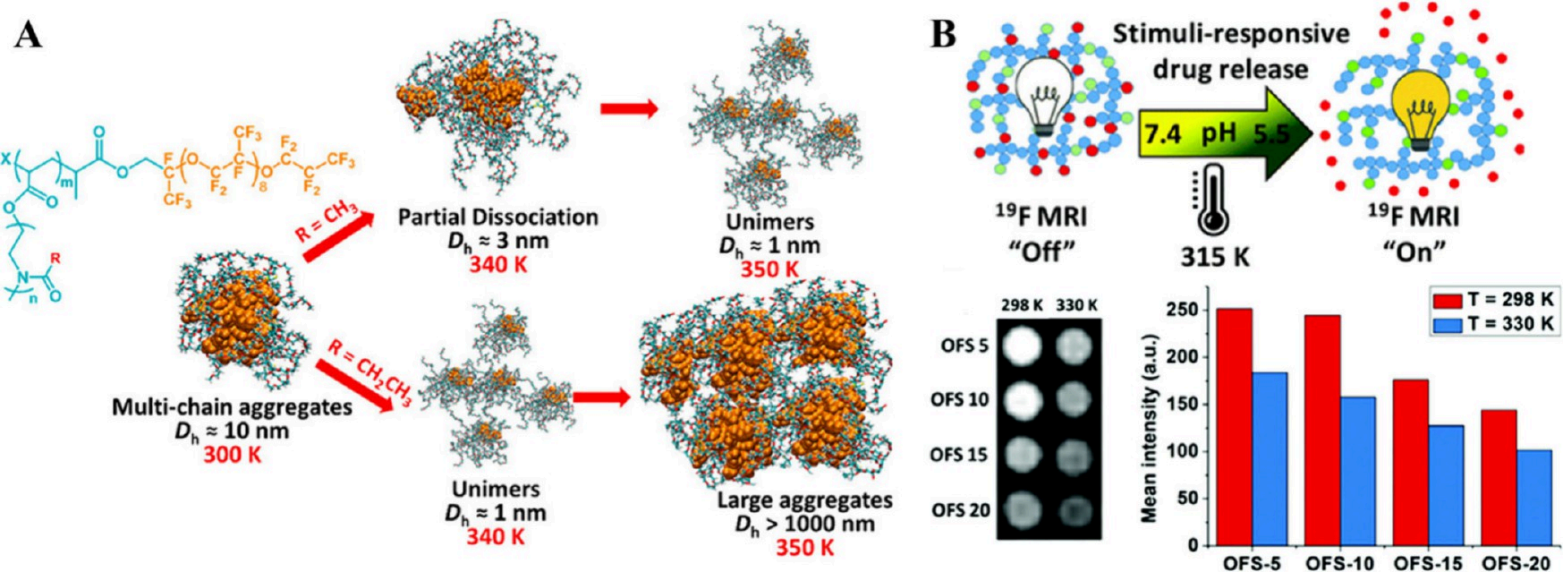
(A) Schematic illustration of the temperature-driven transition of bottlebrush-like poly(2-oxazoline)-b-PFPE aggregates between 300 and 350 K. Reproduced with permission from ref (46). Copyright 2019, American Chemical Society. (B) Schematic illustration of the stimuli-responsive drug release, ^19^F MR images, and signal intensities of TFEA-containing statistical copolymers. Reproduced with permission from ref (48). Copyright 2021, Royal Society of Chemistry.

Short hydrophobic perfluoropolyethers (PFPEs) can be used to construct thermoresponsive block copolymers with a hydrophilic block consisting of bottlebrush-shaped poly(2-oxazoline)s, as reported by Whittaker et al. (Figure 6A).^46^ At room temperature, these copolymers self-assembled into multichain aggregates. With the increase in temperature, the aggregates dissociated into unimers, and their ^19^F MRI intensity increased. Further heating above the LCST of the poly(2-oxazoline) brush-block resulted in macroscopic aggregation and ^19^F MRI signal attenuation. Similar findings have been reported by Kaberov and colleagues for triblock copolymers containing hydrophilic, thermoresponsive, and fluorinated poly(2-oxazoline) blocks.^47^

Usman and colleagues have also designed a dual stimuli-responsive switchable ^19^F MR tracer (Figure 6B).^48^ A series of free statistical copolymers were prepared from thermoresponsive oligoethylene glycol methyl ether methacrylate (OEGMA), fluorinated 2,2,2-trifluoroethyl acrylate (TFEA), and hydrophobic styrene, which was used to adjust the LCST of the polymer to physiological temperatures. Increasing the temperature of the environment weakened the ^19^F signal and resulted in peak splitting, because the fluorines experienced two different environments. However, all the polymers were visualized, even at temperatures above LCST. Furthermore, these authors synthesized copolymers containing a model drug attached to the monomer with acid-cleavable hydrazone linkage and without styrene units to observe dual stimuli-responsiveness. At acidic pH, hydrazone was cleaved, and the drug was completely released within 10 h. ^19^F MR images of these polymers at different temperatures were compared under neutral and acidic pHs and at 0 and 48 h. At neutral pH (pH 7.4), where there was no drug release, no change in signal intensity was observed after 48 h at the same temperature, but when the temperature increased above LCST, the signal weakened. At acidic pH, more specifically at pH 5.5, and high temperature, the drug release enhanced the signal intensity by ∼25%.

Through solid-phase peptide synthesis (SPPS), Zhu and colleagues developed thermoresponsive peptide-based, amphiphilic, monodisperse polyethylene glycol (M-PEG) combs modified with fluorinated l-lysine for ^19^F MR imaging.^49^ These combs yielded sharp and finely tuned LCSTs with the help of their structural accuracy. For ^19^F NMR experiments, the polymer with an LCST of 31 °C was selected and analyzed at temperatures ranging from 15 to 35 °C. Due to the enhanced dipolar interactions between fluorines, peak broadening was observed at increased temperatures, but the signal remained clearly detectable. Furthermore, the same group also assessed the effect of different M-PEG sizes, geometries, and PEGylation sites on the physicochemical and biological properties to understand the structural benefits.^50^ With their multifunctional probe design, prepared by fluorescent N-terminal addition to the peptidic M-PEG comb having fluorinated l-lysine moieties, they also demonstrated the tunability of this system.^51^

In addition to thermoresponsive systems, pH-responsive ^19^F MRI tracers have been reported, as well. For example, Criscione and colleagues prepared pH-responsive, partly fluorinated poly(amidoamine) (PAMAM) dendrimers, which self-assembled into nanoscopic and macroscopic particulates.^52^ After the covalent attachment of perfluoroalkyl moieties to primary amines of the dendrimer, the densely packed dendrimers self-assembled in water due to fluorophobic interactions. These assemblies were further tested in the delivery of small molecule cargos, for which Rhodamine B was selected as a model molecule. At low pH, upon repulsion caused by protonation of the remaining free amines, these particulates disassembled over time, releasing the encapsulated cargo. Cargo release at pH 2.0 was two and four times faster than that at pH 5.0 and 7.0, respectively, while the fluorine signal intensity remained virtually unchanged.

pH-responsive cleavage of a hydrophobic moiety from a nanogel core can improve segmental mobility and amplify the ^19^F MRI signal. These improvements were reported by Munkhbat and colleagues, who developed fluorinated nanogels responsive to pH via their acid-degradable tetrahydropyranyl (THP) moieties.^53^ To synthesize the polymeric nanogel, they prepared statistical copolymers of hydrophilic polyethylene glycol monomethyl ether acrylate (PEGA), cross-linkable pyridyl disulfide ethyl acrylate (PDSA), acid-cleavable THP acrylate, and fluorinated 2,2,2-trifluoroethyl methacrylate (TFEMA) via reversible addition–fragmentation chain transfer (RAFT) copolymerization, followed by cross-linking. In an acidic environment, THP moieties were hydrolyzed while preserving the size and composition of the nanogel. However, the *T*_2_ relaxation times increased sharply due to the increased mobility of ^19^F nuclei, thereby enhancing ^19^F MR signal intensity compared to nonhydrolyzed nanogels. *In vivo*^19^F MRI of nanogels showed strong signals in inflammation sites of mouse models of chronic inflammation. The authors also demonstrated that these systems can be applied as theranostics by encapsulating a chemotherapeutic drug molecule (docetaxel, DTX), albeit without testing their therapeutic effect in animal models.

Concurrent tandem polymerization (CTP) is a method whereby monomer or end-group modification reactions orthogonally overlap with polymerizations based on controlled radical polymerizations (CRPs), such as atom transfer radical polymerization (ATRP) and RAFT polymerization. Based on RAFT polymerization, Fu and colleagues developed a CTP system in which trifluoroethyl methacrylate (TFEMA) monomers were in situ transformed into different alcohol-based methacrylate monomers in an enzymatic transacylation reaction with alcohols during polymerization.^54^ Using this system, they prepared multifunctional copolymers with fluorinated groups for ^19^F imaging, azido groups for further click reactions, aldehyde groups for modifications with amine-containing molecules, and poly(ethylene glycol) (PEG) for enhanced water solubility. Through a copper-catalyzed azide–alkyne cycloaddition (CuAAc) click reaction, they modified the azido groups on the polymer chain using the glucose molecule as a representative targeting molecule to assess the ^19^F imaging potential of the resulting polymer by measuring fluorine signals under preclinical conditions. Then, they attached an amine-containing chemotherapy drug (DOX) to the aldehyde groups of the polymers via pH-sensitive imine bonds, which are known to hydrolyze under acidic conditions. After these modifications, the amphiphilic polymer self-assembled into a micelle. Even though the DOX release was faster at acidic pH than at neutral pH, these authors did not perform ^19^F imaging with the sample containing DOX. So, whether micelle formation with DOX attachment or release affected fluorine signal intensity remains unknown.

### 3.3 Stimuli-Responsive Nonself-Assembled Fluorinated Polymers

Stimuli-responsiveness can be leveraged to trigger drug release in non-self-assembling polymer systems. Such systems are stable and tunable without any phase change or fluorine signal attenuation, enabling us to follow the distribution of the tracer. The stimuli-responsive release of a hydrophobic drug can lead to ^19^F MRI signal amplification by increasing the chain hydration and mobility of a hydrophilic polymer. Fuchs and colleagues prepared switchable hyperbranched polymers (HBPs) for theranostics using trifluoroethyl acrylate (TFEA) as a fluorinated moiety of HBP synthesized by RAFT polymerization.^55^ Subsequently, they attached hydrophobic drugs to the polymer backbone via either acid- (hydrazone) or redox-cleavable (disulfide) bonds. Stimuli-responsive drug release was induced by an acidic pH (pH 5) or by adding a reductive (tris(2-carboxyethyl) phosphine hydrochloride agent. Upon drug release, the *T*_2_ relaxation time increased with the increase in fluorine mobility, and the ^19^F MR signal intensity was enhanced by approximately 30% for a pH-cleavable system and 50% for a redox-responsive system. Notwithstanding this signal amplification, using such a system as an “on–off” switch may require further structure optimization to enhance signal amplification under biological conditions. The advantage of this proposed method lies in drug release quantification based on the increase in the signal intensity.

**Figure 7:**
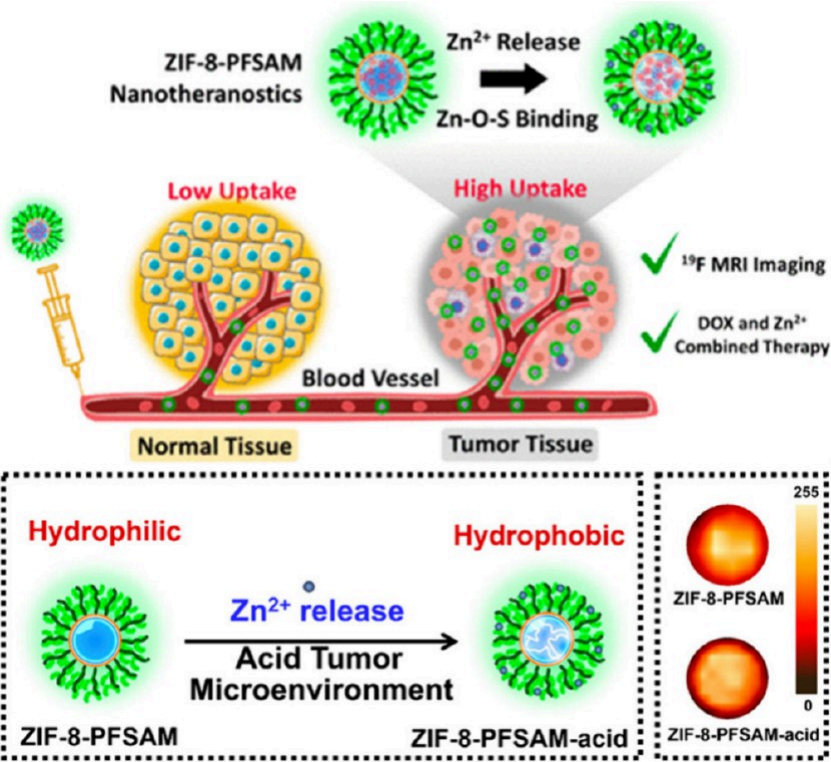
Schematic illustration of the cancer imaging with pH-responsive nanoparticles accumulated in the tumor tissue, triggered Zn^2+^/DOX release, and *in vitro*^19^F MRI of nanoparticles. Reproduced with permission from ref (57). Copyright 2023, American Chemical Society.

Metal–organic frameworks (MOFs) represent emerging materials that are popular for their high porosity and synthetic tunability.^56^ By using their advantages, Wang and colleagues synthesized nanoparticles based on zeolitic imidazolate framework-8 (ZIF-8), composed of zinc ions and 2-methylimidazole (MIM), conjugated with sulfoxide containing fluorinated thermoresponsive homopolymer, poly(*N*-(2-((2,2,2-trifluoroethyl)sulfinyl)-ethyl)acrylamide) (PFSAM) (Figure 7).^57^ Applying the grafting-from strategy, they synthesized PFSAM directly on ZIF-8 by surface-initiated RAFT polymerization. The structure was further functionalized by encapsulating DOX, which is known for its synergistic chemotherapeutic effect with Zn^2+^. These nanoparticles were stable in body pH and had strong ^19^F signals. However, in an acidic environment, ZIF-8 degraded and released Zn^2+^ while maintaining the ^19^F signal intensity. Its authors further proved their concept in an *in vivo* experiment conducted in mice, whose ^19^F MR imaging showed that nanoparticles with ZIF-8 and PFSAM provided excellent tumor targeting. Nevertheless, the thermoresponsive feature of PFSAM was overlooked in this study. More recently, Emrick and colleagues developed a new water-soluble fluorinated monomer structurally similar to FSAM, but with zwitterionic sulfobetaine (SB) moieties.^58^ Although they obtained homo- and copolymers with narrow dispersities, the redox responsiveness of these polymers has not yet been published.

Local tissue oxygenation is a biomarker for the diagnosis and monitoring of some diseases, including cancer.^59^ Paramagnetic O_2_ shortens the *T*_1_ relaxation time of fluorine and enhances ^19^F performance.^60^ These effects were previously explored with perfluorocarbon (PFC)-encapsulated nanoparticles. To understand the effectiveness of water-soluble oxygen-sensing probes, Taylor and colleagues synthesized fluorinated copolymers from monomers with a high density of chemically equivalent fluorines (2,2,2-trifluoroethyl acrylate (TFEA), hexafluorooxyethylacrylate (HexaFOEA), and nonafluorooxyethylacrylate (NonaFOEA)) by RAFT polymerization.^61^ Poly(ethylene glycol) acrylate (PEGA) was selected as the hydrophilic comonomer to enhance water solubility. Both HexaFOEA and NonaFOEA had stronger fluorine signals than TFEA, but NonaFOEA displayed the highest signal intensity with a signal-to-noise ratio (SNR) three times higher than that of TFEA under similar fluorine compositions. HexaFOEA-containing polymers showed the lowest *T*_1_ times, which is helpful for *T*_1_-weighted imaging. Regardless of their composition, each copolymer series containing the same fluorinated monomer had similar *T*_1_ values. Across all the copolymers, *T*_2_ decreased with the increase in fluorine content due to the increased restriction on fluorine mobility.

The authors also followed the variation of the *T*_1_ relaxation times (or relaxivity, the inverse value of *T*_1_) with the partial pressure of oxygen (pO_2_) to evaluate the ^19^F signal sensitivity to pO_2_. A calibration curve of pO_2_ versus relaxivity (inverse of *T*_1_) was prepared. They found a strong correlation between the wt % of fluorine and the oxygen sensitivity of the samples. Although the system must still be improved for clinically relevant conditions, their water-soluble probes rival recently published PFC-loaded nanoparticles, indicating their potential for noninvasive oxygen monitoring.^62^

High ionic concentrations are other biomarkers for cancerous tissues as these contain high concentrations of sodium and chlorine, in contrast to healthy tissues, so their ionic compositions can be used for detection.^63^ Leveraging this difference, Zhang and colleagues investigated the ionic effect on the thermoresponsiveness of oligo(ethylene glycol)-methacrylate (OEGMA)-based polymers to improve the design of stimuli-responsive ^19^F tracers.^64^ For this purpose, they prepared a random copolymer of the OEGMA and 2,2,2-trifluoroethyl acrylate (TFEA) by RAFT polymerization and found that the LCST of polymer solutions strongly depended on the type and concentration of the salt in solution. Only in the presence of salt was the LCST lower than 80 °C. In pure water, LCST was higher.

In doing so, these authors were the first to use a fluorinated monomer to adjust the LCST of OEGMA, which is also beneficial for the future design of new stimuli-responsive probes for ^19^F MRI. However, the mechanism of interaction between salts and polymers remained unknown until the same research group published a detailed study on the impact of the conformation and mobility of these ion-responsive fluorinated copolymers, poly(OEGMA-co-TFEA), in the presence of salts, applied for the detection of normal and cancer cells.^65^ The study involved both ^19^F NMR and molecular dynamics (MD) simulations. As shown by dynamic light scattering (DLS) measurements, the addition of NaCl decreased polymer sizes, suggesting stronger intramolecular interactions resulting from the formation of an ionic hydration layer around the polymer and weaker hydrogen bonding. By ^1^H NMR nuclear Overhauser effect spectroscopy (^1^H NOESY), they found that the decrease in the hydrodynamic size of the copolymer in the presence of NaCl was stemming from OEGMA side chain aggregation. MD simulations aligned with DLS and ^1^H NOESY experiments, and ^19^F NMR results indicated a decrease in *T*_2_ relaxation times upon NaCl addition due to partial dehydration of the polymers and closer interactions between TFEA units. When analyzing the polymer in pure water and healthy and cancer cells, they identified a decrease in *T*_2_ relaxation times upon shifting the polymer environment from pure water (178 ms) to normal cells (124 ms, due to the presence of ions), and the *T*_2_ relaxation times further decreased in cancer cells (82 ms, higher ion content). Based on these findings, the authors suggested that the *T*_2_ relaxation time difference can be used as a marker for cancer cells.

The pH changes can also be used as biomarkers to follow the difference between the physiological pH and slightly acidic pH of tumors, inflammation sites, and endolysosomes. Gianolio and colleagues prepared a fluorinated supramolecular tracer for pH mapping via ratiometric ^19^F and ^1^H MRIs. Poly-β-cyclodextrin (poly-β-CD), having 8 to 10 β-CD units, was used to guest gadolinium (Gd^3+^)-containing and fluorine-containing adamantanes.^66^ The authors suggested that paramagnetic Gd units were far enough from fluorinated units to avoid any signal reduction by the PRE effect. pH responsiveness was provided by the Gd-containing unit, which was deprotonated at the basic pH and protonated at the acidic pH. With the help of ^1^H relaxivity changes, pH mapping was obtained *in vitro*, and ^19^F was used as a reporter to normalize Gd concentrations.

## 4 Stimuli-Responsive Fluoropolymer Tracers with “Off-On” MR Signal Switching

Significant ^19^F MRI signal amplification or attenuation upon internal stimuli enables the noninvasive detection of changes in pH, reactive oxygen species (ROS), and specific enzymes often associated with diseases such as cancer or inflammation. In stimuli-responsive fluoropolymers, ^19^F MRI signal intensity mostly varies due to changes in fluorinated chain mobility or the presence of a paramagnetic ^19^F MRI quencher. In this section, we provide an overview of such “off-on” polymer systems (Figure 1B, Table 2) in which the signal intensity at least doubles (amplification).

**Table 2:** Overview of Stimuli-Responsive ^19^F MR Tracers with “Off-On” Signal Switching

stimulus	responsive unit	activation mechanism	^19^F MR response	ref
pH	PDEAMA	Swelling of fluorinated core	OFF → ON	(68)
pH	PDMAEMA	Swelling of nanoparticles	OFF → ON	(71)
pH	PDMAEMA	Conformational changes	OFF → ON	(72)
pH	LDH	Disappearance of PRE effect	OFF → ON	(73)
Redox	DNBS	Disassembly	OFF → ON	(78)
Redox	Disulfide	Branched to linear polymers	Amplification	(79)
Redox	α-Ketoamide	Release of fluorines	OFF → ON	(80)
Enzyme	Peptide	Drug release	OFF → ON	(81)
Enzyme	Ester	Release of fluorines	OFF → ON	(82)
Enzyme	Ester	Disassembly	OFF → ON	(82)
ROS + pH	Disulfide + DPAEMA	Disassembly	OFF → ON	(83)
ROS/pH	End-caps	Depolymerization	OFF → ON	(85)
ROS + light	Disulfide + ICG	Disassembly	OFF → ON	(86)
ROS	PFTAM	Disassembly	Shift	(91)
Redox	Ferrocene	Disassembly	Shift	(92)
HP-β-CD	AdamCF_3_A	Host–guest interactions	Shift + amplification	(93)
pH	Secondary amines (PDBA/PDPA/PC6A)	Disassembly	Multi-OFF → ON	(94)

### 4.1 pH-Responsive Fluoropolymer Tracers with “Off-On” Switching

Changes in pH are arguably the most universal and widely studied stimuli.^67^ pH-responsive polymer systems are usually applied to exploit the difference between the physiological pH of 7.4 and the slightly acidic environment of tumors, inflammation sites, and endolysosomes. Most often, the polymer structure contains hydrophobic tertiary amine units. These units become positively charged at low pH values, which leads to their hydrophilization. Alternatively, a hydrophobic moiety (e.g., drug) linked to a hydrophilic polymer via an acid-degradable linker can be released at low pH values, resulting in hydrophilization of the whole system.

In 2007, Oishi and colleagues developed the first smart pH-sensitive nanosized ^19^F tracers based on PEGylated nanogels containing pH-sensitive polyamine cores and fluorinated moieties synthesized by emulsion copolymerization.^68,69^ Tertiary amine-containing 2-(*N,N*-diethylamino)ethyl methacrylate (DEAMA) in the nanogel core was the pH-responsive unit, while 2,2,2-trifluoroethyl methacrylate (TFEMA) was introduced in different mole percentages as the fluorinated unit. At a physiological pH of 7.4, the gel collapsed, so no ^19^F NMR signal was observed because the nanogel core was hydrophobic due to the deprotonated amino groups of DEAMA. This failure to detect any ^19^F NMR signal was confirmed by the meager *T*_2_ relaxation times. However, when the pH decreased upon DEAMA protonation, the gel core swelled, activating the ^19^F signal. As a result, the *T*_2_ values increased, owing to the higher mobility of the fluorines.

Similarly, Wang and colleagues prepared pH-responsive semifluorinated core-cross-linked star polymer nanoparticles.^70^ In their design, the tertiary amino-containing 2-(dimethylamino)ethyl methacrylate (DMAEMA) monomer was used as a pH-responsive modality, whereas 2,2,2-trifluoroethyl acrylate (TFEA) was the fluorinated comonomer with its three identical fluorine-containing −CF_3_ groups. Star polymers were prepared by RAFT polymerization with a branched core containing TFEA, DMAEMA, and, as a cross-linker, ethylene glycol dimethacrylate (EGDMA) and a hydrophilic shell consisting of poly(ethylene glycol) methyl ether methacrylate (PPEGMA) brushes. The ^19^F signals of these star nanoparticles exhibited a strong dependence on the pH due to the tertiary amines. When the pH dropped below the p*K*_a_ of the DMAEMA unit, the tertiary amines became protonated, which created an electrostatic repulsion between the charged chains, so the nanoparticles were swollen. In this way, fluorinated units were highly mobile and well separated from each other and had long *T*_2_ relaxation times. Conversely, increasing the pH above the p*K*_a_ of the DMAEMA unit induced amine deprotonation and core shrinking, decreasing both the mobility and *T*_2_ relaxation times. In this study, they showed that the ^19^F NMR peak was intense in the pH range from 4 to 7.4. However, when the pH was increased above 7.4, they observed significant fluorine NMR peak attenuation and broadening. The same research group also prepared star polymer nanoparticles in which the core was cross-linked with a redox-responsive disulfide group containing bis(2-methacryloyl)oxyethyl disulfide (DSDMA), with similar pH-responsiveness and added disulfide for biodegradability.^71^ However, the ^19^F signal intensity with respect to GSH and disulfide breakage was not reported in the study.

Further employing a pH-responsive amine monomer switching strategy, Zalewski and colleagues prepared semifluorinated copolymers of 2,2,2-trifluoroethyl methacrylate (TFEMA) or 1,1,1,3,3,3-hexafluoroisopropyl methacrylate (HFiPMA) with 2-(dimethylamino)ethyl methacrylate (DMAEMA), and 2-hydroxyethyl methacrylate (HEMA) via ATRP.^72^ Thanks to the tertiary amine structure of DMAEMA, these structures were pH-responsive. When decreasing the pH, they observed that ^19^F MR signal intensity increased for both TFEMA and HFiPMA. For TFEMA, the main difference was noted in *T*_2_ times, which increased with the increase in the TFEMA content and with the decrease in pH, whereas *T*_1_ times were only slightly affected. For HFiPMA, the lowest *T*_1_ was detected at pH 8, and this parameter gradually increased at both lower and higher pH values, whereas the *T*_2_ times rapidly decreased with the pH up to the range of 7–8.

**Figure 8:**
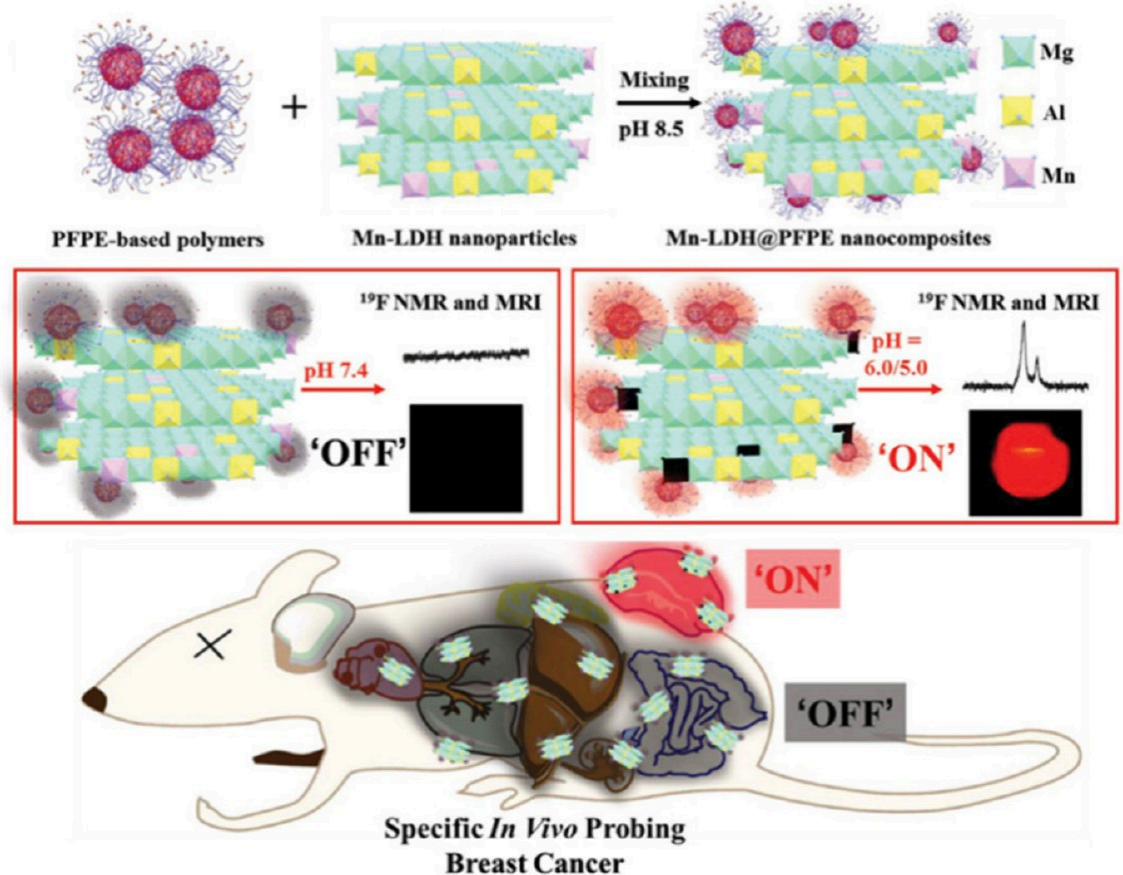
Schematic illustration of the chemical design and *in vivo*/*in vitro* pH-response of fluoropolymer-containing nanocomposites. Reproduced with permission from ref (73). Copyright 2019, John Wiley and Sons.

Layered double hydroxides (LDHs) are known to dissolve in acidic environments and, as such, are useful materials for preparing pH-responsive biomaterials. Zhang and colleagues developed a pH-activated system using manganese-doped LDH nanoparticles (Mn-LDH) and acrylic acid (AA) containing oligo(ethylene glycol) methyl ether acrylate (OEGA) copolymers with a perfluoropolyether (PFPE) macro-chain transfer agent (macroCTA) (Figure 8).^73^ With the help of AA segments, the negative charges on the synthesized polymer chains attracted Mn-LDHs, forming pH-responsive nanocomposites. At nonacidic pHs, the ^19^F MR signal was quenched due to the paramagnetic relaxation enhancement (PRE) effect caused by paramagnetic manganese, which decreased the relaxation times of fluorine nuclei. In an acidic environment, Mn^2+^ was leached from the nanocomposite. Therefore, the PRE effect on the fluorine ceased, which turned the ^19^F signal “on”, creating an intense and detectable signal during *in vitro* and *in vivo* measurements. When these nanoparticles were coated with bovine serum albumin (BSA) protein to prevent aggregation and applied to a tumor-bearing mouse, the ^19^F signal was distinctively detectable in the tumor region. In healthy tissue, only some ^19^F signal artifacts were detected.

### 4.2 Redox-Responsive Fluoropolymer Tracers with “Off-On” Switching

Redox-responsive polymers have gained substantial attention in biomacromolecular research.^74^ In particular, systems responding to reactive oxygen species (ROS) highly prevalent in hypoxic tumors and inflamed sites are widely studied these days.^75^ As for pH-responsive systems, two main strategies have been used to construct ROS-responsive polymers—either oxidation of hydrophobic repeating units (e.g., hydrophobic thioether to hydrophilic sulfoxide or sulfone)^76^ or ROS-triggered cleavage of the hydrophobic moiety.^77^

**Figure 9:**
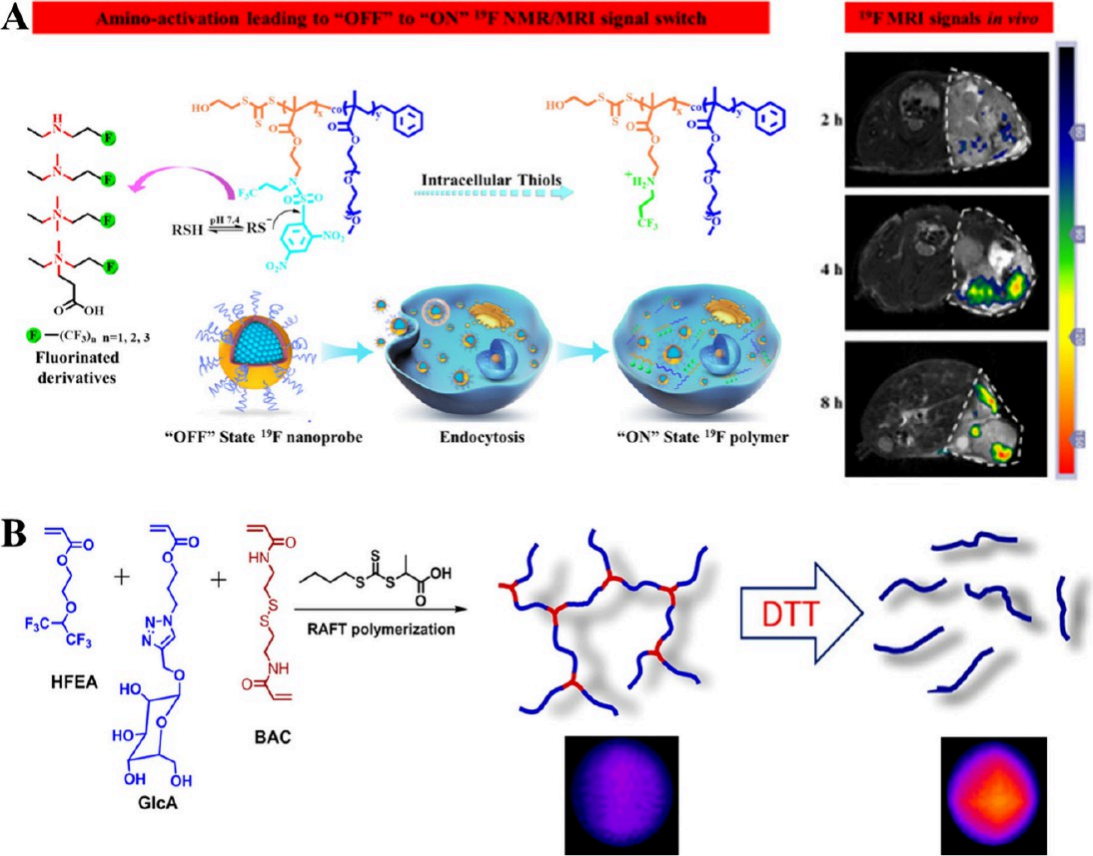
Redox-responsive fluoropolymers with “off-on” ^19^F MRI activation. (A) Schematic illustration of the intracellular reducing microenvironment-induced amino-activatable fluorinated probes and *in vivo*^19^F MR images of biothiols. Reproduced with permission from ref (78). Copyright 2018, American Chemical Society. (B) Synthesis of branched polymer and schematic illustration of the DTT reduction with *in vitro*^19^F MR images. Reproduced with permission from ref (79). Copyright 2019, American Chemical Society.

The ability to trigger the cleavage of a hydrophobic substituent was demonstrated by Huang and colleagues, who synthesized a ^19^F MRI probe responsive to the intracellular reducing microenvironment (Figure 9A).^78^ This ^19^F MRI probe can be used to trace biothiols (e.g., glutathione) by the selective cleavage of a 2,4-dinitrobenzenesulfonamide (DNBS) group. To prepare this probe, the authors synthesized copolymers of fluorinated 2-((2,4-dinitro-*N*-(3,3,3-trifluoropropyl)-phenyl)sulfonamido)-ethyl methacrylate (AMA-DNBS-F) and hydrophilic poly(ethylene glycol) methyl ether methacrylate (mPEGMA) by RAFT polymerization followed by nanoprecipitation, yielding nanoprobes.

This nanoprobe had tightly packed fluorines in its hydrophobic core, which restricted mobility, shortened the *T*_2_ relaxation time, and silenced the ^19^F signal. However, in contact with thiolated molecules, the DNBS groups of AMA-DNBS-Fs were removed by nucleophilic substitution. With a disrupted hydrophobic core, the nanoprobe disassembled, thus increasing the *T*_2_ relaxation times and recovering the ^19^F signal. In selectivity tests performed with various biological analytes, the ^19^F signal was detected only in the presence of thiols, including cysteine and reduced glutathione. By *in vivo*^19^F MR imaging of biothiols, tumor tissues were visualized in mouse models. Therefore, this cellular thiol-responsive feature may promote early diagnosis by imaging of specific biomarkers.

Fluoropolymer backbone degradation can amplify ^19^F MRI signals by increasing the segmental chain mobility. Such ^19^F MRI signal amplification was exemplified by Fu and colleagues when preparing branched fluorinated glycopolymers by random RAFT polymerization of d-glucose glycomonomer (GlcA), a newly developed fluorinated monomer (2-((1,1,1,3,3,3-hexafluoropropan-2-yl)oxy)ethyl acrylate (HFEA)), and cross-linking monomer *N,N′*-bis(acryloyl)cystamine (BAC), which is responsive to reduction through its disulfide linkage (Figure 9B).^79^ The cross-linked glycopolymers provided strong ^19^F signals and showed no *in vitro* cytotoxicity. Moreover, reductive species changed the polymer topology from a branched to a linear structure, thus increasing the *T*_*2*_ relaxation and SNR by increasing structural mobility. Cellular uptake experiments revealed that the synthesized probe was successfully internalized by cells through carbohydrate–protein interactions owing to the biological recognition of repeating carbohydrate units of glycopolymers.

Li and colleagues developed a water-soluble, peroxynitrite-responsive, self-assembled fluorinated nanoprobe (PSAP) with cleavable-fluorinated moieties.^80^ PSAP was prepared from a peroxynitrite (ONOO^–^)-responsive fluorinated amphiphilic molecule (PFAM), which was composed of a hydrophilic PEG tail and two peroxynitrite-responsive fluorinated hydrophobic arms. Due to their amphiphilicity, they self-assembled in an aqueous solution, forming nanoparticles with a highly packed fluorinated core. The restricted core mobility reduced the *T*_2_ relaxation times and switched the ^19^F signal off. When PSAP was in contact with ONOO^–^, the nanoprobe was disassembled by releasing a small fluorinated molecule. This disassembly increased fluorine mobility, enhanced the *T*_2_ relaxation time, and recovered the ^19^F signal. Furthermore, PSAP was nonresponsive to pH changes and selectively responsive to ONOO^–^, as shown by ^19^F measurements in various biologically relevant molecules, including ROS. Because ONOO^–^ is a drug-induced liver injury (DILI) biomarker, *in vivo* experiments were performed in mice with DILI (and, hence, ONOO^–^ in the liver). ^19^F detection in the liver of living mice demonstrated that this tracer is suitable for deep-tissue real-time imaging of ONOO^–^.

### 4.3 Enzymatically Responsive Fluoropolymer Tracers with “Off-On” Switching

Enzymatic response was also studied for off-on ^19^F MRI systems. An example has been developed by Alhaidari and Spain to monitor drug release kinetics.^81^ Hyperbranched hydrophilic poly(*N*,*N*-dimethylacrylamide) (HB-PDMA) has been synthesized by RAFT polymerization for such a purpose. 5-Fluorouracil (5-FU) was selected as a model fluorinated drug and attached to the polymer backbone via an enzyme-responsive peptide linker. When the probe was exposed to the S9 fraction, a mixture containing metabolizing enzymes, 5-FU was released, which increased fluorine mobility and the *T*_2_ relaxation time. Fluorine signal intensity was directly proportional to the amount of drug released, and only 9% of drug release was achieved in this study. Although the drug release should be enhanced for high-quality ^19^F signals, the research is promising for the detection of attached and released drugs separately.

**Figure 10:**
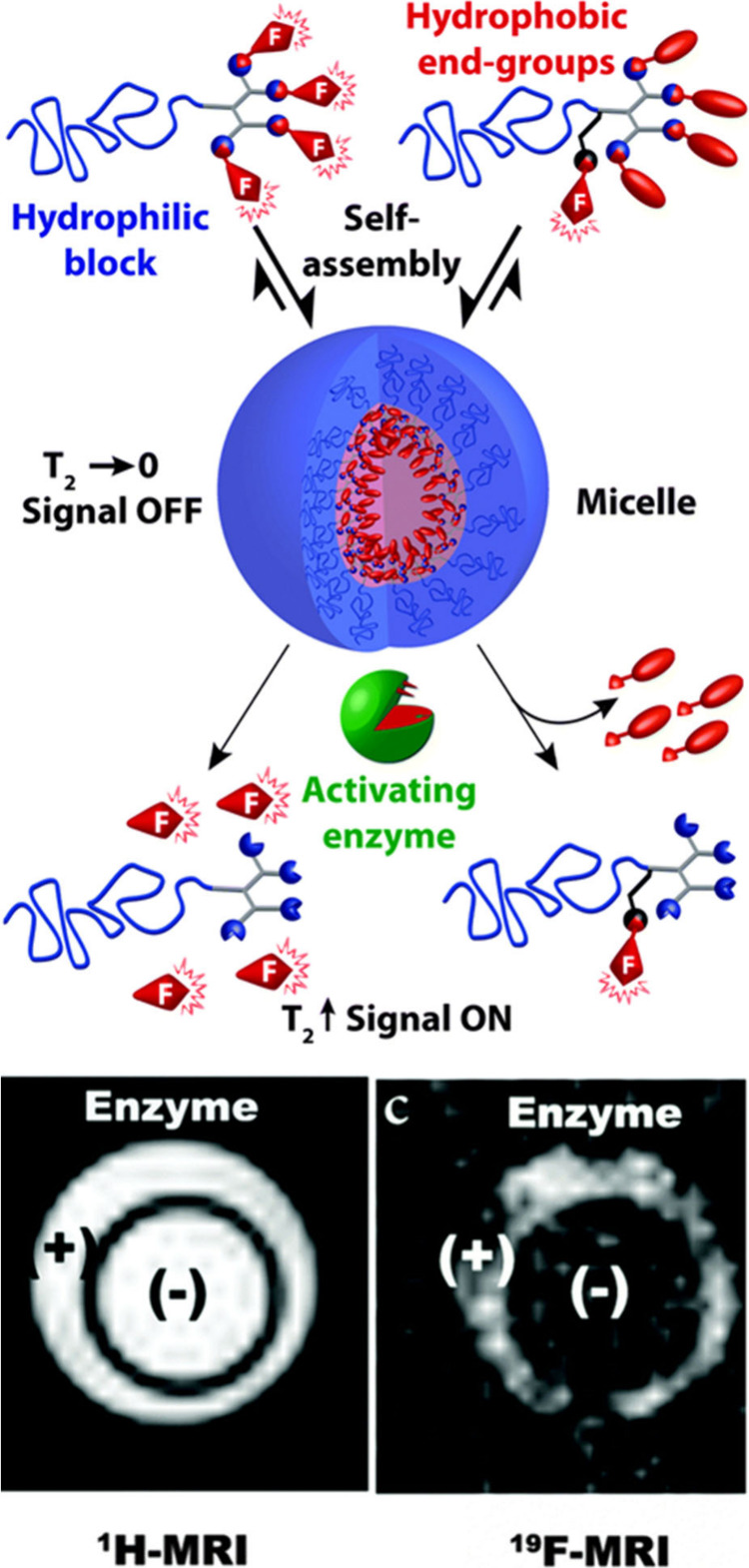
Enzyme-responsive fluoropolymers with “off-on” ^19^F MRI activation by cleavage of a fluorinated end-group and its ^1^H/^19^F MR images. Reproduced with permission from ref (82). Copyright 2016, Royal Society of Chemistry.

Similar to the previously explained peroxynitrite-responsive tracer design (in Section 4.2) by Li et al.,^80^ having cleavable fluorine moieties, enzyme-responsive smart micellar probes of PEG-dendron hybrids were developed by Buzhor and colleagues and were labeled either with cleavable or noncleavable fluorinated groups (Figure 10).^82^ For cleavable labeling, fluorine-containing end-groups were attached with a cleavable ester linkage. For noncleavable labeling, fluorine units were attached to the backbone via an amide linkage, and a hydrophobic nonfluorinated group was attached to the polymer chain end via an ester linkage. In the presence of porcine liver esterase (PLE), an ester-cleaving enzyme, small fluorinated molecules were released in the cleavable approach, creating a strong signal. However, during their study, the authors found that fluorines might interfere with the enzymatic activity, slowing the reaction. Nevertheless, cleaving the hydrophobic end groups disassembled the micelles and provided a strong ^19^F signal enhanced by the increase in *T*_2_ relaxation time. By contrast, the ^19^F signal was weaker in the noncleavable approach due to a smaller number of fluorines, but the cleavage kinetics were faster in this method.

### 4.4 Switchable Fluoropolymer Tracers Combining Multiple Responses

Combining multiple responses into a single system may further enhance the potential of fluoropolymer tracers. As an example, Fu and colleagues combined ROS- with pH-responsiveness to enhance ^19^F MRI sensing.^83^ First, they synthesized a ROS-responsive polymer by ATRP starting from a poly(ethylene glycol)-based hydrophilic macroinitiator (PEG-Br). The macroinitiator was chain-extended by statistical copolymerization of the thioether-containing stimuli-responsive monomer 2-((2-((2-(ethylthio)ethyl)thio)ethyl)thio)ethyl methacrylate (ETEMA) and the fluorinated monomer 2,2,2-trifluoroethyl methacrylate (TFEMA). The block copolymer self-assembled into a structure with a stimuli-responsive fluorinated compact core with a ^19^F MR signal switched “off” due to a very short *T*_2_ relaxation time. Upon exposure to H_2_O_2_ (used as a model ROS), the thioether was oxidized to sulfoxide, disrupting the hydrophobicity of the core and leading to the disassembly of the structure. A well-known feature, this disassembly increased fluorine mobility and the *T*_2_ relaxation times, thus switching “on” the ^19^F MR signal. However, these results were achieved under very high concentrations of H_2_O_2_. At lower concentrations, thioether conversion decreased, so the future development of more sensitive probes may require structure optimization.

To advance their system, these authors synthesized a dual-responsive probe with both ROS- and pH-responsiveness by adding a 2-(diisopropylamino)ethyl methacrylate (DPAEMA) monomer to the copolymerization mixture. In other words, this probe leveraged the pH-responsiveness of tertiary amines, as discussed in a previous chapter. Thanks to their additional hydrophilicity, when exposed to both ROS and acidic pH, these dual-responsive polymers provided a much more enhanced ^19^F signal than when exposed to just an acidic environment or an ROS. Such probes with dual-responsive features may open up opportunities to increase imaging sensitivity.

Self-immolative polymers (SIP) are a class of polymers that undergo irreversible head-to-tail cascade depolymerization.^84^ With fluorinated moieties on the side chains of the polymers, Ding and colleagues prepared an amphiphilic self-immolative block copolymer.^85^ These micelles self-immolated into small molecule derivatives of azaquinone methides (AQMs) triggered by acidic pH or ROS depending on the end-cap. At neutral pH and in nonreductive environments, molecular motions were restricted by the self-assembled structure, decreasing the *T*_2_ relaxation times and suppressing the ^19^F MR signal. However, depolymerization occurs when self-immolation by pH or ROS is triggered depending on the end-cap. Under such circumstances, *T*_2_ times increased, and ^19^F signals were recovered. Moreover, the incorporation of DOTA-Gd moieties also showed that self-immolative ^19^F/^1^H dual probes might be used as ratiometric ROS detection probes.

**Figure 11:**
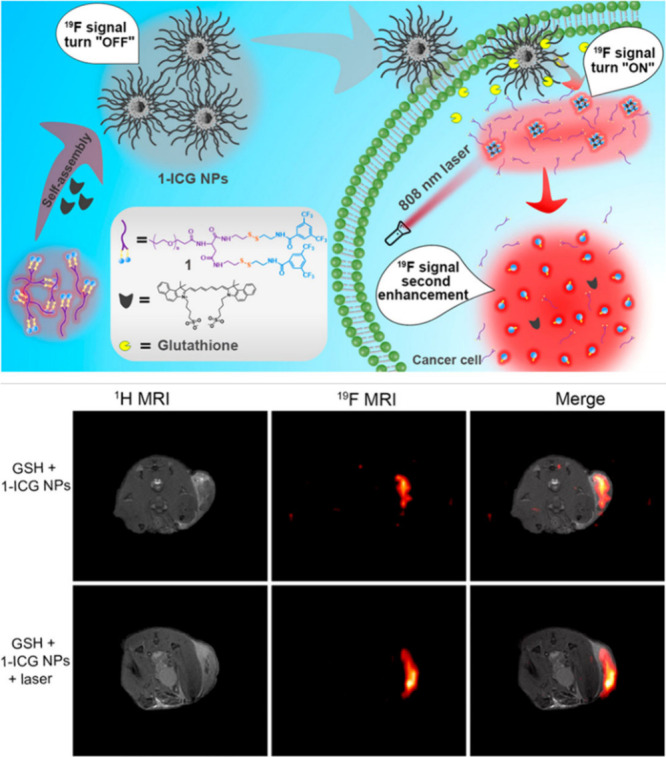
Combination of light and redox responsiveness in a multistimuli-responsive ^19^F nanotracers with two-step signal activation/amplification and their *in vivo*^19^F MR images. Reproduced with permission from ref (86). Copyright 2020, American Chemical Society.

Tang and colleagues combined light and redox responsiveness in a multistimuli-responsive ^19^F nanoprobe (Figure 11).^86^ Without any stimuli, the ^19^F signal was “off” because the amphiphilic polymer with fluorinated moieties attached to redox-responsive disulfide groups was self-assembled. This probe was designed for a two-step signal amplification. Upon disulfide linker cleavage by GSH, abundant in cancer tissue, the ^19^F signal was activated. This signal was then amplified upon the complete disassembly of the nanoparticles under NIR light, which also had a photothermal therapy effect enhanced by heating, resulting from indocyanine green (ICG) molecules. In this study, two-step ^19^F signal activation and amplification were shown in both cell and animal experiments.

## 5 “Multicolor” Stimuli-Responsive Fluoropolymers with Chemical Shift

Altering the chemical environment of ^19^F nuclei often leads to a change in a ^19^F MR spectrum, which can be employed in stimuli-responsive multispectral probes. The probes with spectrally distinct ^19^F MR peaks are often termed “multicolor” or “color-coded”, as nuclei with different resonance frequencies can be visualized independently by different colors.^87^ The presence of a reactive group in close proximity to the fluorinated group can lead to a stimuli-responsive change in the ^19^F MR chemical shift upon reaction. In the case that both the starting compound and product show distinct peaks differing in the chemical shift, one can monitor the reaction kinetics in complex biological media. Compared to the above-mentioned probes with ^19^F MR signal attenuation or amplification, no internal reference has to be used to account for the probe concentration change. Such stimuli-responsive ^19^F MRI multicolor probes are widely studied, mainly for small molecule tracers (e.g., PFCEs, fluorinated ionic liquids),^88−90^ but stimuli-responsive multicolor fluoropolymer tracers have gained attention in recent years, as well (Figure 1C).

**Figure 12:**
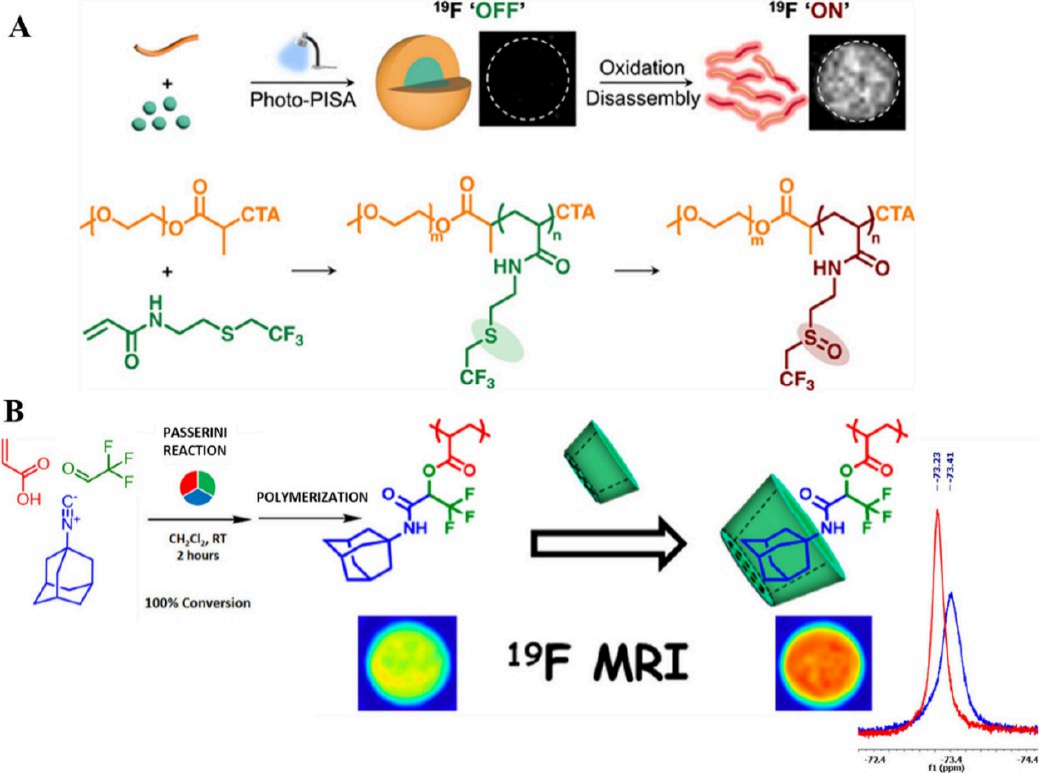
“Multicolor” stimuli-responsive fluoropolymers that show a change in the ^19^F MR signal shift upon activation. (A) Oxidation-responsive thioether-containing nanotracers prepared by photo-PISA and their ^19^F MR response to disassembly. Reproduced with permission from ref (91). Copyright 2024, American Chemical Society. (B) Adamantane-containing fluoropolymers that show a ^19^F MR signal shift upon host–guest interaction with β-cyclodextrin. Reproduced with permission from ref (93). Copyright 2019, American Chemical Society.

ROS-responsive oxidation of thioether to sulfoxide can induce a change in the ^19^F MR chemical shift of the proximal fluorinated group. For example, Chang and colleagues have developed oxidation-responsive fluorinated nanoparticles by polymerization-induced self-assembly (PISA) (Figure 12A).^91^ They synthesized polymers using a methoxy polyethylene glycol (mPEG)-based macro-chain transfer agent (macroCTA) and a new monomer *N*-(2-((2,2,2-trifluoroethyl)thio)ethyl)-acrylamide (FTAM) through photoinduced electron/energy transfer reversible addition–fragmentation chain transfer (PET-RAFT) polymerization.

FTAM is a fluorinated thioether-containing monomer that is soluble in a water–ethanol mixture. However, its polymer PFTAM is insoluble in the same solvent. Therefore, TFAM was utilized as a core-forming monomer in PISA, starting from water-soluble macroCTA. When the FTAM polymerization proceeded, the fluorinated units self-assembled into a nanoparticle core, whereas PEG units of macroCTA formed a hydrophilic shell. Owing to strong interactions between fluorines in the micellar core, the mobility of ^19^F atoms was highly restricted, which suppressed the ^19^F MR signal. On the other hand, the presence of oxidants (e.g., ROS) leads to the oxidation of thioethers to more hydrophilic sulfoxide groups. As a result, the nanoparticles disassembled, increasing the mobility of fluorinated segments and thus generating a fluorine signal.

Furthermore, the oxidation triggered a change in the ^19^F MR chemical shift from a nonoxidized thioether to sulfoxide. Even though the represented system requires high concentrations of oxidative agents and long reaction times for the oxidation of PFTAM units, these nanoparticles may prove effective in the ratiometric detection of oxidative species in living systems with the help of signal shifts after the oxidation. Such systems can then be used for the imaging of hypoxic tumors or inflammation sites due to their high ROS concentrations compared to healthy tissues.

Oxidation of hydrophobic ferrocene (Fe^2+^) to more hydrophilic ferrocenium (Fe^3+^) by ROS can lead to a change in the ^19^F MR shift of adjacent fluorinated groups. Švec and colleagues synthesized redox-responsive amphiphilic poly(2-oxazoline) copolymers by cationic ring-opening polymerization (CROP) and modified them with trifluoromethylated ferrocene moieties.^92^ In an aqueous environment, the copolymers formed nanoparticles that can be potentially used for hydrophobic drug encapsulation as well. These nanoparticles were redox-responsive due to their ferrocene moieties in the structure. In the reduced state, fluorinated ferrocenes (Fe^2+^) are diamagnetic. Through oxidation, they are converted into positively charged paramagnetic ferrocenium ions (Fe^3+^), which trigger the disassembly of nanoparticles by increasing the core hydrophilicity and changing the chemical shift and relaxation times of the fluorine nuclei. *In vitro*, the reduced and oxidized polymers were visualized separately and showed no cytotoxicity, but *in vivo* studies have not yet been published.

Host–guest interactions are another class that may yield a change of chemical shift, as explained by Couturaud and colleagues (Figure 12B).^93^ They synthesized a bifunctional monomer, trifluorinated adamantyl acrylate (AdamCF_3_A), that has fluorines for imaging and adamantane units for supramolecular binding. By the Passerini reaction between acrylic acid, 1-adamantyl isocyanide, and trifluoroacetaldehyde, the monomer was prepared in one batch without byproducts. Then, they synthesized a water-soluble copolymer containing AdamCF_3_A and monitored relaxivity changes and fluorine signal intensities with respect to (2-hydroxypropyl)-β-cyclodextrin (HP-β-CD) host–guest interactions. After the supramolecular binding of HP-β-CD, two different *T*_2_ relaxation times were obtained depending on the proximity of fluorines to the polymer backbone (major contribution to the fluorine signal) or to the host–guest site. It has been shown that after supramolecular binding the mobility of fluorines and their signal intensities increased, and a signal shift of 0.2 ppm was observed. While providing a basis for the development of fluorinated supramolecular probes, the work has the potential for monitoring the success of drug delivery systems by *in vivo*^19^F MR imaging.

**Figure 13:**
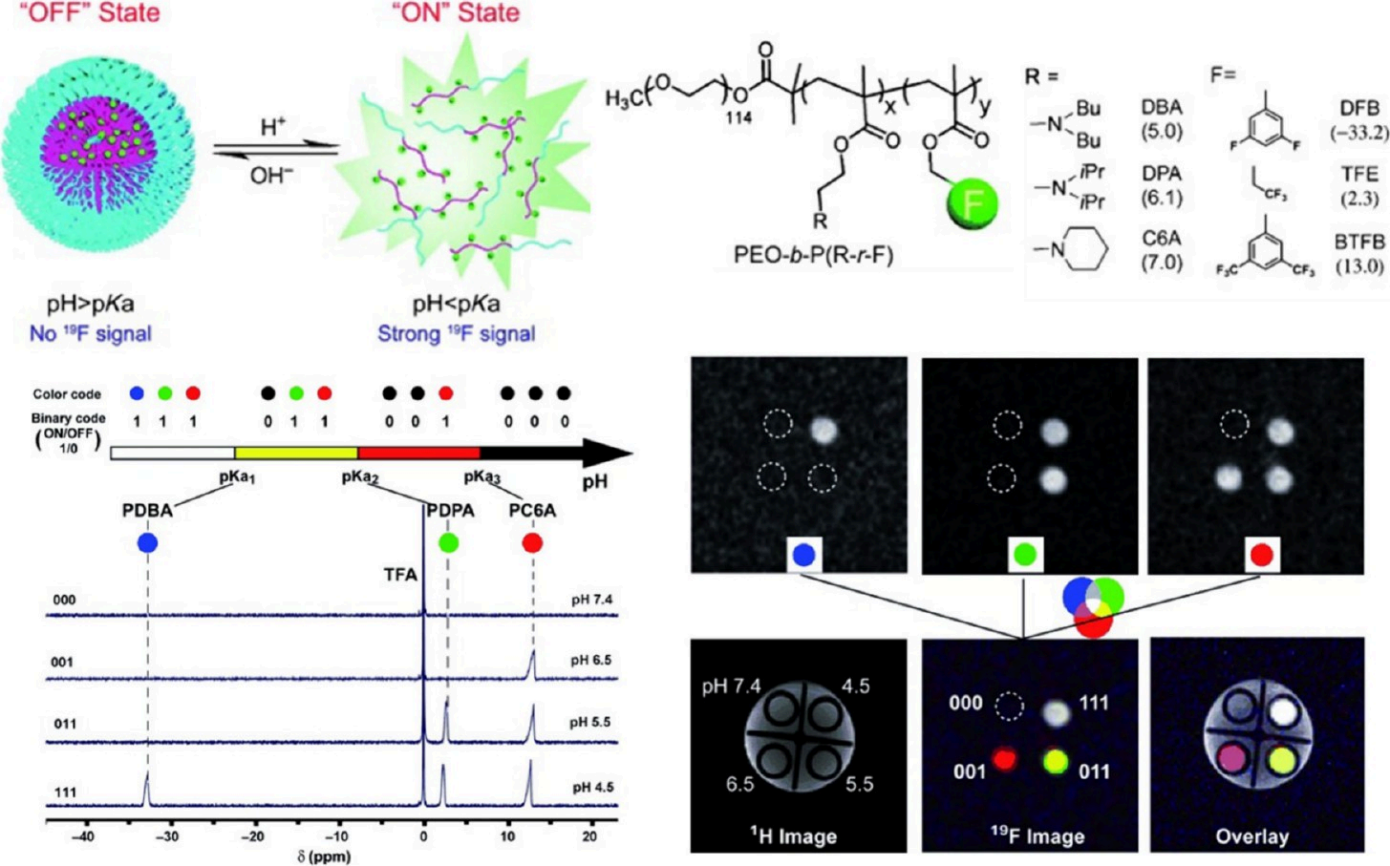
“Multicolor” pH-responsive fluoropolymers that show a change in ^19^F MR signal shift upon triggered disassembly with a depiction of the experimental design for multicolor imaging. Reproduced with permission from ref (94). Copyright 2013, John Wiley and Sons.

Huang and colleagues designed a different system for multicolored imaging (Figure 13).^94^ Rather than inducing a chemical reaction on the molecule to cause a chemical shift on the fluorine signal, they prepared multiple compounds with different chemical shifts at different environmental pHs. More specifically, these authors developed “multicolored” pH-responsive “off/on” nanoprobes from ionizable diblock copolymers capable of detecting narrow pH transitions. With these nanoprobes, each pH range was labeled with a specific ^19^F signal shift. For proof of concept, they synthesized three nanoprobes consisting of two different blocks. In all nanoprobes, the first block was a hydrophilic poly(ethylene oxide) (PEO) to enhance water solubility, but the second block varied with each structure to ensure a sensitive response to pH changes. Therefore, in their designs, each nanoprobe had a different pH-responsive tertiary amine segment (with different p*K*_a_ values) and a different fluorinated segment (with specific chemical shifts).

When the pH was higher than the p*K*_a_ of the tertiary amine, the copolymers self-assembled in water, forming micelles. These micelles reduced the mobility of fluorines and suppressed the ^19^F signal. Lowering the pH disassembled the micelles and thus promoted fluorine mobility, leading to strong signal activation. The nanoprobes were mixed for a multicolor response; therefore, when the fluorine signals were measured at different pHs, each polymer with a p*K*_a_ value higher than the pH of the environment yielded a signal. In their phantom studies, these researchers demonstrated that pH changes between 7.4, 6.5, 5.5, and 4.5 were detectable with this system.

## 6 Conclusions and Future Perspectives

In this review, we provide an extensive overview of recently published research in the field of stimuli-responsive fluorinated polymers and their application as ^19^F MRI tracers. These polymers have recently emerged as promising materials for advanced diagnostics. Fluoropolymer MRI tracers combine the benefits of ^19^F MRI, such as noninvasiveness and the absence of background signal and potentially toxic metals, with the broad tunability of stimuli-responsive polymers, whose structure can be precisely tailored for targeted applications (e.g., sensing). In some cases, stimuli-responsive polymers have been labeled with fluorine atoms to trace their biodistribution simply. However, an increasing number of reports have focused on switchable ^19^F MRI tracers for which changes in the fluoropolymer microenvironment induce a change in the ^19^F MR signal amplitude or chemical shift. Such switchable tracers can then be used to diagnose specific diseases, such as cancer. Individual visualization of fluorine atoms with different chemical shifts opens up numerous possibilities for developing advanced probes in which, e.g., one fluorine type can be used to trace fluoropolymer biodistribution. In contrast, the other fluorine adjacent to a stimuli-responsive group can then report changes in the internal microenvironment. In particular, tracers showing a triggered change of chemical shift are becoming particularly popular as they allow simultaneous visualization of both signals (before and after the switch). Therefore, they are often called multicolor probes.

While pH-responsive and thermoresponsive fluoropolymer tracers have been widely studied for over a decade, an increasing number of reports on redox-responsive (in particular ROS-responsive) systems have been published in recent years, focusing on specific environments related to diseases, such as cancer or inflammation. In the future, substantial research will likely be conducted on small-molecule-activable ^19^F MRI probes that respond to specific biomarkers. As the concentration of such biomarkers in the body is often low, ^19^F MRI signal amplification methods will be necessary to overcome the relatively low sensitivity of ^19^F MRI. For example, highly sensitive systems where interactions with a single molecule lead to the activation of a high number of fluorines by ceasing paramagnetic quenching or nanoparticle disassembly can “amplify” the MRI response.

Notwithstanding these advancements, the field of fluoropolymer-based ^19^F MRI tracers is still relatively incipient and faces several shortcomings that pose considerable challenges for future research. ^19^F MRI tracers are less sensitive than gadolinium-based contrast agents, as shown by the relatively high concentrations of tracers that must be administered and the extremely long acquisition times, particularly when rapid tracer dilution in the blood pool is expected (i.e., intravenous administration). Nevertheless, rapid advances in MRI hardware should significantly improve the imaging sensitivity of ^19^F MRI tracers by increasing magnetic field strengths and optimizing radiofrequency coils in clinical scanners. Therefore, careful optimization of tracer structures to balance fluorine content, imaging sensitivity, and biocompatibility is the key to developing highly effective ^19^F MRI tracers.

In the field of theranostics, stimuli-responsive fluoropolymer tracers show a high potential for combining polymer therapeutics with diagnostics. In addition to tracing the biodistribution of stimuli-responsive drug delivery systems (for which other suitable imaging can also be used), ^19^F MRI can provide valuable information on triggered drug release kinetics, especially when using “multicolor” switchable polymers. ^19^F MRI/MRS can be used to accurately measure the drug release rate, particularly in comparison with fluorescence-based techniques such as fluorescence lifetime imaging or Förster resonance energy transfer, both of which suffer from low penetration of light through tissues. Furthermore, polymer carrier degradation can be analogously studied by a switchable ^19^F MRI.

Addressing the relatively low sensitivity of ^19^F MRI will be a crucial quest for the use of fluoropolymer tracers in clinical practice. Once this problem is solved, we will be able to exploit the full potential of stimuli-responsive fluoropolymer tracers, particularly for *in vivo* applications. We can then expect extensive research on advanced stimuli-responsive systems with a relatively lower fluorine content such as fluoropolymer-coated inorganic nanomaterials and fluoropolymer–protein conjugates. Nevertheless, the exceptionally high fluorine contents and outstanding ^19^F MRI properties of low-*T*_g_ fluoropolymers (especially PFPE) may be leveraged to develop novel PFPE-based stimuli-responsive tracers. This field has not yet been thoroughly explored.
